# Synthesis and Anti-HIV-1 Activity Evaluation for Novel 3a,6a-Dihydro-1*H*-pyrrolo[3,4-*c*]pyrazole-4,6-dione Derivatives

**DOI:** 10.3390/molecules21091198

**Published:** 2016-09-08

**Authors:** Guan-Nan Liu, Rong-Hua Luo, Yu Zhou, Xing-Jie Zhang, Jian Li, Liu-Meng Yang, Yong-Tang Zheng, Hong Liu

**Affiliations:** 1 College of Life Sciences, China Jiliang University, Hangzhou 310018, Zhejiang, China; gnliu@cjlu.edu.cn; 2 CAS Key Laboratory of Receptor Research, Shanghai Institute of Materia Medica, Shanghai Institutes for Biological Sciences, Chinese Academy of Sciences, Shanghai 201203, China; zhouyu@mail.shcnc.ac.cn (Y.Z.); jianl@mail.shcnc.ac.cn (J.L.); 3 Key Laboratory of Animal Models and Human Disease Mechanisms of Chinese Academy of Sciences and Yunnan Province, Kunming Institute of Zoology, Chinese Academy of Sciences, Kunming 650223, Yunnan, China; luorh@mail.kiz.ac.cn (R.-H.L.); kmhz123@163.com (X.-J.Z.); lmyang@mail.kiz.ac.cn (L.-M.Y.)

**Keywords:** anti-HIV-1, integrase, synthesis, SAR, AIDS

## Abstract

The search for new molecular constructs that resemble the critical two-metal binding pharmacophore and the halo-substituted phenyl functionality required for HIV-1 integrase (IN) inhibition represents a vibrant area of research within drug discovery. As reported herein, we have modified our recently disclosed 1-[2-(4-fluorophenyl)ethyl]-pyrrole-2,5-dione scaffolds to design 35 novel compounds with improved biological activities against HIV-1. These new compounds show single-digit micromolar antiviral potencies against HIV-1 and low toxicity. Among of them, compound **9g** and **15i** had potent anti-HIV-1 activities (EC_50_ < 5 μM) and excellent therapeutic index (TI, CC_50_/EC_50_ > 100). These two compounds have potential as lead compounds for further optimization into clinical anti-HIV-1 agents.

## 1. Introduction

**Figure 1:**
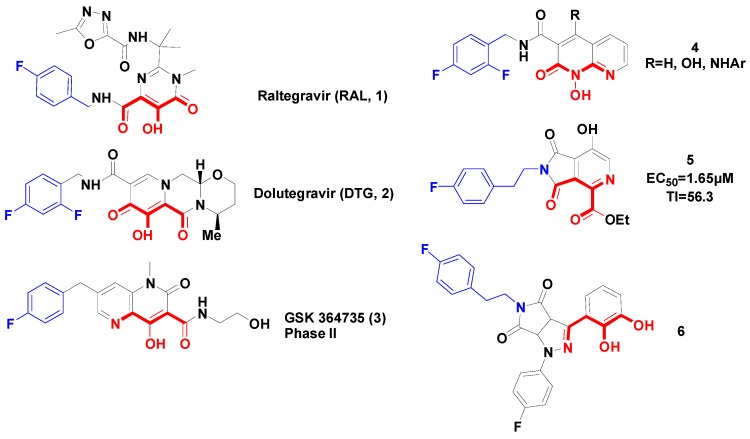
HIV-1 integrase inhibitors.

Human immunodeficiency virus type 1 (HIV-1) is the main causative agent of acquired immunodeficiency syndrome (AIDS), which remains a serious public health problem throughout the world [1]. HIV-1 integrase (IN) is a virally encoded enzyme essential for virus replication, which mediates insertion of the double-stranded DNA provirus into the host genome [2]. Integration is the final step before irreversible and productive HIV-1 infection of the target cell [3]. Although it was identified as an attractive target nearly 20 years ago [4], the first generation drugs targeting IN (raltegravir, RAL, **1** and elvitegravir, ELV) were only recently approved by the Food and Drug Administration (FDA) [5,6] as part of combination antiretroviral therapy (Figure 1). Unfortunately, continuous mutation of the viral genome leads to multi-drug resistant viral strains that are no longer susceptible to the current therapy [7,8].

As a result there has been a surge of research driving force to address these limitations with significant contributions from both academia and industry [2,9,10]. The current studies have been focused on the identification of new chemical classes that are able to retain the intrinsic potency and structural elements of the bidentate metal-binding pharmacophore (red color) which is essential for strand transfer inhibition and the halo-substituted phenyl rings (blue color) which interact with the penultimate cytosine base near the 3’-end of the viral DNA to prevent the insertion of the viral DNA into the host genome [11,12]. These efforts provide several distinct structural classes. Some second-generation drug targeting IN, such as dolutegravir (DTG, **2**) [13], which has been approved for clinical use, and GSK364735 (**3**) [14,15] is in phase II clinical trials (Figure 1). Although they display superior characteristics to RAL and EVG, the cross-resistance has still been observed and the subsequent secondary mutations diminish the inhibitory activity [16,17,18]. Therefore, intensified search for IN inhibitors with novel chemical features to overcome the resistance and more investigation of the structure-activity relationship are urgently needed.

Recently, 1-hydroxy-1,8-naphthyridin-2*H*-one-3-carboxamides **4** have been reported to potently inhibit wild-type IN in biochemical assays and show good antiviral efficacies in single-round infection assays of HIV-1 infectivity [19,20] (Figure 1). Importantly, members of these series retain good antiviral potency against a set of mutants resistant to **1** in these latter assays. A considerable feature of **4** is that the carbonyl oxygen of the 2’,4’-difluorobenzyl amide group (blue color), which binds to the penultimate base adjacent to the 3’-end, may not be an obligatory component of the metal-chelating triad (red color). As a consequence, there may be greater flexibility in this region of the molecule than is found with other inhibitors, such as **1**, where the carbonyl oxygen of the halobenzyl amide participates in Mg^2+^ chelation. This flexibility is reminiscent of what is seen with **2**, where the flexibility of the haloamide component is thought to contribute to the ability of **2** to maintain efficacy against certain forms of IN that are resistant to **1**. Simultaneously, we reported that 7-hydroxy-1,3-dioxo-2,3-dihydro-1*H*-pyrrolo[3,4-*c*]pyridine-4-carboxylates such as **5**, show good anti-HIV activities [21] (Figure 1). In the case of **5**, the binding function is served by a 4’-fluorophenyl ethyl group, which is appended at the 1-position of the pyrrole-2,5-dione nucleus. Previous work has shown that the nature and pattern of halogen phenyl substitution can significantly affect the potency of IN inhibitors [22]. In the discovery of compound **5**, we found that a 4’-fluorophenyl moiety, which is also present in **1** and **3**, was superior to the other halophenyl rings we tested. As a continuation of our research in this field, we further modified the 1-[2-(4-fluorophenyl)ethyl]-pyrrole-2,5-dione nucleus by incorporating a new 2-(2,3-dihydroxyphenyl)-4,5-dihydropyrazole at the 3- and 4-positions to form a new component of the metal-chelating triad (red color) independent of the halogen phenyl functionality (blue color). Additionally, we noted that an extra aryl group in compound **4** can increase their inhibitory potency [20]. Therefore, we have designed compound **6** which has two 4’-fluorophenyl moieties and a novel bidentate metal-binding pharmacophore (Figure 1). Subsequently, we prepared a series of analogues **6**–**16** using an iterative process of design, synthesis, biological evaluation, and redesign.

## 2. Results and Discussion

### 2.1. Chemical Synthesis

**Scheme 1:**
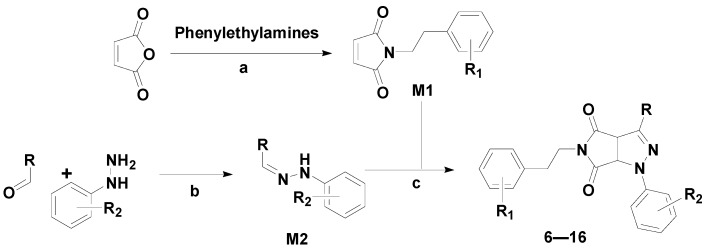
Synthetic route of compounds **6**–**16**. *Reagents and conditions*: (**a**) NaOAc, (Ac)_2_O, reflux, 30 min; (**b**) AcOH, EtOH, room temperature, 8 h; (**c**) EtOH, reflux, 12–20 h.

As outlined in Scheme 1, target compounds **6**–**16** were successfully synthesized from commercially available reagents. In a similar reported methodology, the treatment of maleic anhydride with the corresponding phenylethylamines in acetic anhydride in the presence of sodium acetate gave the intermediate 1-phenylethyl-pyrrole-2,5-diones **M1** [23]. Another intermediaries **M2** was obtained from aryl-aldehydes by treatment of the corresponding phenylhydrazines in ethanol without further purification. Subsequently, **M1** were reacted with **M2** in refluxing ethanol via 1,3-dipolar cycloaddition reaction yielding the target compounds **6**–**16** [24]. All the target compounds were characterized by ^1^H-NMR and MS.

### 2.2. Anti-HIV-1 Evaluation

Target compounds **6–16** were evaluated for their inhibitory activity against HIV-1 replication in acutely infected C8166 cells in vitro according to the previously described method [25,26], and AZT was used as a positive control. The assay results of the target compounds are presented in Table 1 and Table 2, expressed as CC_50_, EC_50_ and therapeutic index (TI). Several of these new compounds show single-digit micromolar antiviral potencies against HIV-1 and low toxicity for cultured cells, which in two cases (**9g** and **15i**), results in therapeutic index (TI, CC_50_/EC_50_) of greater than 100.

Using **6** as a starting point, the initial series of compounds was designed to examine the role of aromatic functionality of the *R*-substitution on the nucleus (Table 1). We initiated our work by replacing the 2-, and 3-hydroxy on the phenyl group. Despite of the improved anti-HIV-1 potencies of analogs without the 2-hydroxy (**7a** and **7b**, EC_50_ values of 5.37 and 9.21 μM, respectively), the cytotoxicities were increased, revealing the essentiality of the hydroxy on the 2-position. The removal of the 3-hydroxy gave the compound **8** with a better anti-HIV-1 potency and a comparable cytotoxicity, suggesting the possibility of replacing the hydroxy on the 3-position of the phenyl group. Thus, we carefully continued our structural optimization effort by introducing some other groups into the 3- and other positions of the phenyl group and remaining the hydroxy on the 2-position. Although moving of the hydroxy from 3-position of the phenyl group to 4-position (**9a**) slightly decreased the potency against HIV-1 relative to **6**. The fluorine substituent was tolerated well, both in positions 3 and 5 of the phenyl ring (compounds **9b** and **9c**), with the EC_50_ values of 5.74 μM and 4.21 μM, respectively. When the fluorine atom in position 3 was replaced by a chlorine atom (**9d**) or a nitro group (**9e**), both the activities and TI values decreased. Interestingly, the introduction of the electron-donating groups to the 3-position (giving **9f** and **9g**) enhanced the potencies, particularly when utilizing the methoxy group.

We were gratified to discover that the 2-hydroxy-3methoxyphenyl substituted derivative **9g** was nearly eight times more active than its parent 2,3-dihydroxyphenyl substituted counterpart **6** (EC_50_ values of 3.98 and 26.39 μM, respectively) with a TI of greater than 100. The shift of the methoxy group from position 3 to 4 and 5 gave the compounds **10a** and **10b** with slightly decreased anti-HIV-1 activities (EC_50_ values of 8.52 and 4.73 μM, respectively), while replacing the methoxy group on position 3 by ethoxy (compound **10c**) or adding a nitro group to position 5 of the phenyl group (compound **11**) had negative impacts on anti-HIV-1 potency too. Unfortunately, the attempt to replace the hydroxy group in position 2 by electron-donating groups (compounds **12a** and **12b**) led to the decreased potencies. We sequentially continued our structural optimization effort by introduction some other aryl groups into the R group. Good biological activity was observed when 2-hydroxy-naphthalen-1-yl was introduced into the molecule (**13**, EC_50_ = 4.50 μM), while the introduction of the thiazol-2-yl group led to a decrease in the activity by an order of magnitude (compound **14a**), as well as its substitution with pyridin-2-yl group (compound **14b**).

Encouraged by the improved potency of compound **9g**, the diversities of R_1_ and R_2_ substituents were further investigated (Table 2). We prepared an additional series of analogues **15** in which several substituents were introduced at the R_2_-position. Historically, a halo-substituted moiety in this position has been optimal, but little structural variation was tolerated in this series.

Both the shift of the fluorine atom from the 4-position to the 2-, and 3-position (**15a** and **15b**), or the replacement of the fluorine atom on 4-position by chlorine atom (**15c**) led to a potency decrease, even with the use of the 2,4-difluoro phenyl group as in **2** and **4** (**15d**). In general, the derivatives with electron-withdrawing groups also provided poor activities against HIV-1 with EC_50_ values >30 μM (**15e**–**15g**).

**15e**–**15**, as well as the unsubstituted derivative **15h**. Luckily, when the methyl group replaced the 4-fluorine atom on the phenyl group the resulting compound **15i** showed comparable potency to **9g** (EC_50_ = 4.10 μM, TI > 103.09), while the introduction of the 4-methoxy (**15j**) had a negative impact on HIV-1 inhibition. Finally, a diversity of R_1_ substituents has been screened (series **16**), which led a slightly decrease of the antiviral activities, with EC_50_ values of more than 10 μM. An exception was the 2-fluoro substituted derivative **16a**, which provided the best potency (EC_50_ = 2.52 μM) among all the series, but also the highest cytotoxicity.

## 3. Experimental Section

### 3.1. Chemistry 

#### 3.1.1. General Information

All commercially available compounds and solvents were used without further purification. All target products were characterized by their ^1^H-NMR and MS spectra. ^1^H-NMR spectra were recorded in deuterochloroform (CDCl_3_) on a Bruker AMX-300 NMR spectrometer (Bruker Cor., Zurich, Switzerland). Chemical shifts were reported in parts per million (ppm, δ) downfield from tetramethylsilane. Proton coupling patterns are described as singlet (s), doublet (d), triplet (t), quartet (q), multiplet (m), and broad (br). Low- and high-resolution mass spectra (LRMS and HRMS) were measured on Finnigan MAT 95 and LCQ-DE-CA mass spectrometers (Thermo Scientific Inc., Waltham, MA, USA).

#### 3.1.2. General Procedure for the Preparation of **M1**

In a 100 mL three-necked flask provided with a stirrer, a reflux condenser, and a dropping funnel were placed maleic anhydride (0.98 g, 10 mmol) and diethyl ether (25 mL). The maleic anhydride dissolved upon stirring, at which point a solution of the corresponding phenylethylamines (10 mmol, 1 equiv) in diethyl ether (5 mL) was run through the dropping funnel. The resulting thick suspension was stirred at room temperature for 1 h and was then cooled in an ice bath, filtered and dried. Subsequently, the residue was added to a flask containing a solution of anhydrous sodium acetate (0.33 g, 4 mmol) in acetic anhydride (5 mL) and stirred under reflux for 30 min. The reaction mixture was then cooled to room temperature in a cold water bath and was then poured into an ice-water mixture (30 mL). The precipitated product was recovered by filtration, washed three times with portions 10 mL of ice-cold water, and dried as white to yellow solids. The physical data of **M1** have been reported recently [21].

#### 3.1.3. General Procedure for the Preparation of **M2**

To a solution of aryl-aldehydes (5.0 mmol) and the corresponding phenylhydrazines (5.0 mmol) in ethanol (25 mL), was added one drop of acetic acid. The reaction mixture was stirred at room temperature for 8 h. After removing of the solvent, the residue was dissolved in CH_2_Cl_2_ (DCM) (20 mL), washed by water (10 mL), dried over Na_2_SO_4_, filtered and concentrated to give the hydrazones **M2** that were used without further purification.

#### 3.1.4. General Procedure for the Preparation of Target Compounds **6**–**16**

A solution of hydrazone **M2** (0.40 mmol) and **M1** (0.40 mmol) in EtOH was heated at reflux for 12–20 h. After cooling, the solvents were removed. The residue was subjected to a column chromatography on silica gel with petroleum ether (PE)/EtOAc (4/1, *v/v*) as eluent to afford the target compounds **6**–**16**.

*3-(2,3-Dihydroxyphenyl)-1-(4-fluorophenyl)-5-[2-(4-fluorophenyl)ethyl]-3a,6a-dihydro-1H-pyrrolo[3,4-c]pyrazole-4,6-dione* (**6**): Pale yellow solid; ^1^H-NMR (CDCl_3_) δ 7.44–7.38 (m, 3H), 7.13–6.99 (m, 5H), 6.93 (t, *J* = 7.5 Hz, 1H), 6.83 (t, *J* = 8.4 Hz, 2H), 4.99–4.88 (m, 2H), 3.83 (t, *J* = 6.9 Hz, 2H), 2.94 (t, *J* = 7.2 Hz, 2H) ppm; MS (EI, *m/z*) 463 [M]^+^; HRMS (EI) *m/z* calcd C_25_H_19_F_2_N_3_O_4_ [M]^+^ 463.1344, found 463.1341.

*1-(4-Fluorophenyl)-5-[2-(4-fluorophenyl)ethyl]-3-(3-hydroxy-2-methoxyphenyl)-3a,6a-dihydro-1H-pyrrolo[3,4-c]pyrazole-4,6-dione* (**7a**): Pale yellow solid; ^1^H-NMR (CDCl_3_) δ 7.54 (d, *J* = 8.7 Hz, 1H), 7.50–7.45 (m, 2H), 7.06–7.00 (m, 4H), 6.95 (t, *J* = 8.1 Hz, 2H), 6.47–6.43 (m, 2H), 5.30 (d, *J* = 10.5 Hz, 1H), 4.89 (d, *J* = 10.8 Hz, 1H), 3.88 (s, 3H), 3.76–3.71 (m, 2H), 2.85 (t, *J* = 6.9 Hz, 2H) ppm; MS (ESI, *m/z*) 478 [M + H]^+^; HRMS (ESI) *m/z* calcd C_26_H_21_F_2_N_3_O_4_Na [M + Na]^+^ 500.1398, found 500.1400.

*1-(4-Fluorophenyl)-5-[2-(4-fluorophenyl)ethyl]-3-phenyl-3a,6a-dihydro-1H-pyrrolo[3,4-c]pyrazole-4,6-dione* (**7b**): Pale yellow solid; ^1^H-NMR (CDCl_3_) δ 8.02–7.98 (m, 1H), 7.54–7.43 (m, 5H), 7.09–7.00 (m, 4H), 6.84 (t, *J* = 8.7 Hz, 2H), 5.05 (d, *J* = 7.5 Hz, 1H), 4.82 (d, *J* = 7.8 Hz, 1H), 3.81–3.76 (t, *J* = 6.9 Hz, 2H), 2.87 (t, *J* = 8.1 Hz, 2H) ppm; MS (ESI, *m/z*) 430 [M − H]^+^; HRMS (ESI) *m/z* calcd C_25_H_18_F_2_N_3_O_2_ [M − H]^+^ 430.1367, found 430.1369.

*1-(4-Fluorophenyl)-5-[2-(4-fluorophenyl)ethyl]-3-(2-hydroxyphenyl)-3a,6a-dihydro-1H-pyrrolo[3,4-c]pyrazole-4,6-dione* (**8**): Pale yellow solid; ^1^H-NMR (CDCl_3_) δ 7.84–7.81 (m, 1H), 7.44–7.33 (m, 3H), 7.11–6.98 (m, 6H), 6.84 (t, *J* = 8.4 Hz, 2H), 4.98–4.89 (m, 2H), 3.81 (t, *J* = 6.9 Hz, 2H), 2.88 (t, *J* = 7.2 Hz, 2H) ppm; MS (ESI, *m/z*) 446 [M − H]^+^; HRMS (ESI) *m/z* calcd C_25_H_18_F_2_N_3_O_3_ [M − H]^+^ 446.1316, found 446.1327.

*3-(2,4-Dihydroxyphenyl)-1-(4-fluorophenyl)-5-[2-(4-fluorophenyl)ethyl]-3a,6a-dihydro-1H-pyrrolo[3,4-c]pyrazole-4,6-dione* (**9a**): Pale yellow solid; ^1^H-NMR (CDCl_3_) δ 7.72 (d, *J* = 8.4 Hz, 1H), 7.42–7.38 (m, 2H), 7.11–7.00 (m, 4H), 6.88–6.82 (m, 2H), 6.55–6.50 (m, 2H), 4.89–4.88 (m, 2H), 3.86 (t, *J* = 6.9 Hz, 2H), 2.87 (t, *J* = 7.5 Hz, 2H) ppm; MS (EI, *m/z*) 463 [M]^+^; HRMS (EI) *m/z* calcd C_25_H_19_F_2_N_3_O_4_ [M]^+^ 463.1344, found 463.1331.

*1-(4-Fluorophenyl)-5-[2-(4-fluorophenyl)ethyl]-3-(3-fluoro-2-hydroxyphenyl)-3a,6a-dihydro-1H-pyrrolo[3,4-c]pyrazole-4,6-dione* (**9b**): Pale yellow solid; ^1^H-NMR (CDCl_3_) δ 7.60 (d, *J* = 8.9 Hz, 1H), 7.45–7.40 (m, 2H), 7.09–6.95 (m, 6H), 6.84 (t, *J* = 8.7 Hz, 4H), 5.0 (d, *J* = 10.5 Hz, 1H), 4.91 (d, *J* = 11.1 Hz, 1H), 3.83 (t, *J* = 7.2 Hz, 2H), 2.89 (t, *J* = 6.9 Hz, 2H) ppm; MS (ESI, *m/z*) 464 [M − H]^+^; HRMS (ESI) *m/z* calcd C_25_H_17_F_3_N_3_O_3_ [M − H]^+^ 464.1222, found 464.1217.

*1-(4-Fluorophenyl)-5-[2-(4-fluorophenyl)ethyl]-3-(5-fluoro-2-hydroxyphenyl)-3a,6a-dihydro-1H-pyrrolo[3,4-c]pyrazole-4,6-dione* (**9c**): Pale yellow solid; ^1^H-NMR (CDCl_3_) δ 7.69-7.65 (m, 1H), 7.22–7.19 (m, 1H), 7.06–6.92 (m, 7H), 6.87–6.81 (m, 2H), 5.41 (d, *J* = 10.5 Hz, 1H), 4.76 (d, *J* = 9.9 Hz, 1H), 3.75 (t, *J* = 7.5 Hz, 2H), 2.86 (t, *J* = 6.9 Hz, 2H) ppm; MS (ESI, *m/z*) 464 [M − H]^+^; HRMS (ESI) *m/z* calcd C_25_H_17_F_3_N_3_O_3_ [M − H]^+^ 464.1222, found 464.1228.

*3-(3-Chloro-2-hydroxyphenyl)-1-(4-fluorophenyl)-5-[2-(4-fluorophenyl)ethyl]-3a,6a-dihydro-1H-pyrrolo[3,4-c]pyrazole-4,6-dione* (**9d**): Pale yellow solid; ^1^H-NMR (CDCl_3_) δ 7.78–7.75 (m, 1H), 7.46–7.41 (m, 3H), 7.12–6.94 (m, 6H), 6.84 (t, *J* = 8.7 Hz, 2H), 5.02 (d, *J* = 10.8 Hz, 1H), 4.91 (d, *J* = 10.8 Hz, 1H), 3.83 (t, *J* = 6.9 Hz, 2H), 2.89 (t, *J* = 6.9 Hz, 2H) ppm; MS (EI, *m/z*) 481 [M]^+^; HRMS (EI) *m/z* calcd C_25_H_18_ClF_2_N_3_O_3_ [M]^+^ 481.1005, found 481.1016.

*1-(4-Fluorophenyl)-5-[2-(4-fluorophenyl)ethyl]-3-(2-hydroxy-3-nitrophenyl)-3a,6a-dihydro-1H-pyrrolo[3,4-c]pyrazole-4,6-dione* (**9e**): Pale yellow solid; ^1^H-NMR (CDCl_3_) δ 8.13–8.11 (m, 1H), 8.03–7.99 (m, 1H), 7.51–7.47 (m, 2H), 7.11–6.99 (m, 5H), 6.86 (t, *J* = 9.0 Hz, 4H), 5.24 (d, *J* = 11.1 Hz, 1H), 5.05 (d, *J* = 10.8 Hz, 1H), 3.79 (t, *J* = 6.9 Hz, 2H), 2.88 (t, *J* = 6.9 Hz, 2H) ppm; MS (ESI, *m/z*) 491 [M − H]^+^; HRMS (ESI) *m/z* calcd C_25_H_17_F_2_N_4_O_5_ [M − H]^+^ 491.1167, found 491.1171.

*1-(4-Fluoro-phenyl)-5-[2-(4-fluoro-phenyl)-ethyl]-3-(2-hydroxy-3-methyl-phenyl)-3a,6a-dihydro-1H-pyrrolo[3,4-c]pyrazole-4,6-dione* (**9f**): Pale yellow solid; ^1^H-NMR (300 MHz, CDCl_3_) δ 7.69–7.66 (m, 1H), 7.46–7.41 (m, 2H), 7.25–7.22 (m, 1H), 7.11–6.99 (m, 4H), 6.93 (t, *J* = 7.5 Hz, 1H), 6.83 (t, *J* = 8.7 Hz, 2H), 4.98–4.90 (m, 2H), 3.81 (t, *J* = 7.2 Hz, 2H), 2.87 (t, *J* = 7.5 Hz, 2H), 2.35 (s, 3H) ppm; MS (ESI, *m/z*) 460 [M − H]^+^; HRMS (ESI) *m/z* calcd C_26_H_20_F_2_N_3_O_3_ [M − H]^+^ 460.1473, found 460.1476.

*1-(4-Fluorophenyl)-5-[2-(4-fluorophenyl)ethyl]-3-(2-hydroxy-3-methoxyphenyl)-3a,6a-dihydro-1H-pyrrolo[3,4-c]pyrazole-4,6-dione* (**9g**): Pale yellow solid; ^1^H-NMR (CDCl_3_) δ 7.44–7.40 (m, 3H), 7.11–6.96 (m, 6H), 6.88–6.84 (m, 2H), 4.96–4.94 (m, 2H), 3.96 (s, 3H), 3.79 (t, *J* = 7.5 Hz, 2H), 2.87 (t, *J* = 7.2 Hz, 2H) ppm; MS (EI, *m/z*) 477 [M]^+^; HRMS (EI) *m/z* calcd C_26_H_21_F_2_N_3_O_4_ [M]^+^ 477.1500, found 477.1495.

*1-(4-Fluorophenyl)-5-[2-(4-fluorophenyl)ethyl]-3-(2-hydroxy-4-methoxyphenyl)-3a,6a-dihydro-1H-pyrrolo[3,4-c]pyrazole-4,6-dione* (**10a**): Pale yellow solid; ^1^H-NMR (CDCl_3_) δ 7.75–7.72 (m, 1H), 7.42–7.37 (m, 2H), 7.10–6.99 (m, 4H), 6.87–6.82 (m, 2H), 6.59–6.56 (m, 2H), 4.92–4.84 (m, 2H), 3.86–3.78 (m, 5H), 2.87 (t, *J* = 7.5 Hz, 2H) ppm; MS (ESI, *m/z*) 476 [M − H]^+^; HRMS (ESI) *m/z* calcd C_26_H_20_F_2_N_3_O_4_ [M − H]^+^ 476.1422, found 476.1427.

*3-(2,3-Dihydroxyphenyl)-1-(4-fluorophenyl)-5-[2-(4-fluorophenyl)ethyl]-3a,6a-dihydro-1H-pyrrolo[3,4-c]pyrazole-4,6-dione* (**10b**): Pale yellow solid; ^1^H-NMR (CDCl_3_) δ 7.44–7.39 (m, 3H), 7.09–6.96 (m, 6H), 6.84–6.81 (m, 2H), 4.99 (d, *J* = 11.1 Hz, 1H), 4.90 (d, *J* = 10.8 Hz, 1H), 3.86–3.79 (m, 5H), 2.87 (t, *J* = 7.5 Hz, 2H) ppm; MS (ESI, *m/z*) 476[M − H]^+^; HRMS (ESI) *m/z* calcd C_26_H_20_F_2_N_3_O_4_ [M − H]^+^ 476.1422, found 476.1406.

*1-(4-Fluorophenyl)-5-[2-(4-fluorophenyl)ethyl]-3-(3-ethoxy-2-hydroxyphenyl)-3a,6a-dihydro-1H-pyrrolo[3,4-c]pyrazole-4,6-dione* (**10c**): Pale yellow solid; ^1^H-NMR (CDCl_3_) δ 7.46–7.39 (m, 3H), 7.08–6.93 (m, 6H), 6.83 (t, *J* = 8.4 Hz, 2H), 4.98–4.91 (m, 2H), 4.16 (q, 2H), 3.82–3.77 (m, 2H), 2.93–2.83 (m, 2H), 1.51 (t, *J* = 6.9 Hz, 3H) ppm; MS (EI, *m/z*) 491 [M]^+^; HRMS (EI) *m/z* calcd C_27_H_23_F_2_N_3_O_4_ [M]^+^ 491.1657, found 491.1654.

*1-(4-Fluorophenyl)-5-[2-(4-fluorophenyl)ethyl]-3-(2-hydroxy-3-methoxy-5-nitrophenyl)-3a,6a-dihydro-1H-pyrrolo[3,4-c]pyrazole-4,6-dione* (**11**): Pale yellow solid; ^1^H-NMR (CDCl_3_) δ 8.52 (d, *J* = 2.7 Hz, 1H), 7.84 (d, *J* = 2.7 Hz, 1H), 7.45–7.40 (m, 2H), 7.11 (t, *J* = 8.1 Hz, 2H), 7.03–6.98 (m, 2H), 6.81 (t, *J* = 8.4 Hz, 2H), 5.08 (d, *J* = 10.8 Hz, 1H), 4.98 (d, *J* = 11.1 Hz, 1H), 4.05 (s, 3H), 3.85 (t, *J* = 7.8 Hz, 2H), 2.90 (t, *J* = 6.9 Hz, 2H) ppm; MS (ESI, *m/z*) 521 [M − H]^+^; HRMS (ESI) *m/z* calcd C_26_H_19_F_2_N_4_O_6_ [M − H]^+^; 521.1273, found 521.1281.

*3-(2,3-Dimethoxyphenyl)-1-(4-fluorophenyl)-5-[2-(4-fluorophenyl)ethyl]-3a,6a-dihydro-1H-pyrrolo[3,4-c]pyrazole-4,6-dione* (**12a**): Pale yellow solid; ^1^H-NMR (CDCl_3_) δ 7.52–7.47 (m, 2H), 7.26–7.23 (m, 1H), 7.12–6.98 (m, 6H), 6.90 (t, *J* = 8.7 Hz, 2H), 5.23 (d, *J* = 10.8 Hz, 1H), 4.97 (d, *J* = 10.8 Hz, 1H), 3.99 (s, 3H), 3.91 (s, 3H), 3.77–3.71 (m, 2H), 2.85 (t, *J* = 7.2 Hz, 2H) ppm; MS (ESI, *m/z*) 490 [M − H]^+^; HRMS (ESI) *m/z* calcd C_27_H_22_F_2_N_3_O_4_ [M − H]^+^ 490.1578, found 490.1586.

*3-Benzo[1,3]dioxol-4-yl-1-(4-fluorophenyl)-5-[2-(4-fluorophenyl)ethyl]-3a,6a-dihydro-1H-pyrrolo[3,4-c]pyrazole-4,6-dione* (**12b**): Pale yellow solid; ^1^H-NMR (CDCl_3_) δ 7.53–7.49 (m, 2H), 7.36–7.34 (m, 1H), 7.08–7.01 (m, 4H), 6.92–6.85 (m, 4H), 6.09 (s, 2H), 5.02–4.94 (m, 2H), 3.75 (t, *J* = 6.9 Hz, 2H), 2.85 (t, *J* = 7.5 Hz, 2H) ppm; MS (ESI, *m/z*) 474 [M − H]^+^; HRMS (ESI) *m/z* calcd C_26_H_18_F_2_N_3_O_4_ [M − H]^+^ 474.1265, found 474.1259.

*1-(4-Fluorophenyl)-5-[2-(4-fluorophenyl)ethyl]-3-(2-hydroxynaphthalen-1-yl)-3a,6a-dihydro-1H-pyrrolo[3,4-c]pyrazole-4,6-dione* (**13**): Pale yellow solid; ^1^H-NMR (CDCl_3_) δ 7.97 (d, *J* = 2.1 Hz, 1H), 7.83–7.75 (m, 2H), 7.517.39 (m, 4H), 7.176.99 (m, 5H), 6.87 (t, *J* = 8.7 Hz, 2H), 5.55 (d, *J* = 10.8 Hz, 1H), 5.00 (d, *J* = 10.5 Hz, 1H), 3.69 (t, *J* = 7.2 Hz, 2H), 2.80 (t, *J* = 7.5 Hz, 2H) ppm; MS (EI, *m/z*) 497 [M]^+^; HRMS (EI) *m/z* calcd C_29_H_21_F_2_N_3_O_3_ [M]^+^ 497.1551, found 497.1553.

*1-(4-Fluorophenyl)-5-[2-(4-fluorophenyl)ethyl]-3-thiazol-2-yl-3a,6a-dihydro-1H-pyrrolo[3,4-c]pyrazole-4,6-dione* (**14a**): Yellow solid; ^1^H-NMR (CDCl_3_) δ 7.97 (d, *J* = 3.0 Hz, 1H), 7.54–7.50 (m, 2H), 7.40 (d, *J* = 3.0 Hz, 1H), 7.18–7.14 (m, 1H), 7.04–6.97 (m, 3H), 6.85 (t, *J* = 8.4 Hz, 2H), 5.14–5.03 (m, 2H), 3.81 (t, *J* = 6.9 Hz, 2H), 2.88 (t, *J* = 6.9 Hz, 2H) ppm; MS (ESI, *m/z*) 437 [M − H]^+^; HRMS (ESI) *m/z* calcd C_22_H_15_F_2_N_4_O_2_S [M − H]^+^ 437.0884, found 437.0880.

*1-(4-Fluorophenyl)-5-[2-(4-fluorophenyl)ethyl]-3-pyridin-2-yl-3a,6a-dihydro-1H-pyrrolo[3,4-c]pyrazole-4,6-dione* (**14b**): Pale yellow solid; ^1^H-NMR (CDCl_3_) δ 8.75 (m, 1H), 7.98–7.96 (m, 1H), 7.81–7.78 (m, 1H), 7.34–7.32 (m, 1H), 7.21–7.15 (m, 3H), 7.08–6.96 (m, 5H), 5.53 (d, *J* = 10.8 Hz, 1H), 5.14 (d, *J* = 10.8 Hz, 1H), 3.75–3.70 (m, 2H), 2.89-2.84 (m, 2H) ppm; MS (ESI, *m/z*) 431 [M − H]^+^; HRMS (ESI) *m/z* calcd C_24_H_17_F_2_N_4_O_2_ [M − H]^+^ 431.1320, found 431.1318.

*1-(3-Fluorophenyl)-5-[2-(4-fluorophenyl)ethyl]-3-(2-hydroxy-3-methoxyphenyl)-3a,6a-dihydro-1H-pyrrolo[3,4-c]pyrazole-4,6-dione* (**15a**): Pale yellow solid; ^1^H-NMR (CDCl_3_) δ 7.48–7.45 (m, 1H), 7.34–7.13 (m, 3H), 7.04–6.94 (m, 4H), 6.87–6.81 (m, 2H), 6.78–6.72 (m, 1H), 5.03 (d, *J* = 10.8 Hz, 1H), 4.94 (d, *J* = 10.5 Hz, 1H), 3.96 (s, 3H), 3.81 (t, *J* = 7.5 Hz, 2H), 2.87 (t, *J* = 7.2 Hz, 2H) ppm; MS (ESI, *m/z*) 477 [M]^+^; HRMS (ESI) *m/z* calcd C_26_H_21_F_2_N_3_O_4_ [M]^+^ 477.1500, found 477.1489.

*1-(2-Fluorophenyl)-5-[2-(4-fluorophenyl)ethyl]-3-(2-hydroxy-3-methoxyphenyl)-3a,6a-dihydro-1H-pyrrolo[3,4-c]pyrazole-4,6-dione* (**15b**): Pale yellow solid; ^1^H-NMR (CDCl_3_) δ 7.52–7.49 (m, 1H), 7.28–7.26 (m, 1H), 7.19–7.12 (m, 3H), 6.98–6.90 (m, 4H), 6.81 (t, *J* = 8.4 Hz, 2H), 5.41–5.37 (m, 1H), 4.83 (d, *J* = 10.5 Hz, 1H), 3.96 (s, 3H), 3.75 (t, *J* = 6.9 Hz, 2H), 2.81 (t, *J* = 7.2 Hz, 2H) ppm; MS (ESI, *m/z*) 476 [M − H]^+^; HRMS (ESI) *m/z* calcd C_26_H_20_F_2_N_3_O_4_ [M − H]^+^ 476.1422, found 476.1419.

*1-(4-Chlorophenyl)-5-[2-(4-fluorophenyl)ethyl]-3-(2-hydroxy-3-methoxyphenyl)-3a,6a-dihydro-1H-pyrrolo[3,4-c]pyrazole-4,6-dione* (**15c**): Pale yellow solid; ^1^H-NMR (CDCl_3_) δ 7.47–7.30 (m, 4H), 7.18–7.13 (m, 1H), 7.03–6.95 (m, 4H), 6.86–6.81 (m, 2H), 5.00 (d, *J* = 10.7 Hz, 1H), 4.93 (d, *J* = 10.7 Hz, 1H), 3.95 (s, 3H), 3.80 (t, *J* = 6.6 Hz, 2H), 2.87 (t, *J* = 6.9 Hz, 2H) ppm; MS (ESI, *m/z*) 492 [M − H]^+^; HRMS (ESI) *m/z* calcd C_26_H_19_ClFN_3_O_4_ [M − H]^+^ 492.1126, found 492.1120.

*1-(2,4-Difluorophenyl)-5-[2-(4-fluorophenyl)ethyl]-3-(2-hydroxy-3-methoxyphenyl)-3a,6a-dihydro-1H-pyrrolo[3,4-c]pyrazole-4,6-dione* (**15d**): Pale yellow solid; ^1^H-NMR (CDCl_3_) δ 7.51–7.48 (m, 1H), 7.23–7.15 (m, 1H), 7.05–6.78 (m, 8H), 5.25 (d, *J* = 7.8 Hz, 1H), 4.82 (d, *J* = 10.5 Hz, 1H), 3.95 (s, 3H), 3.75 (t, *J* = 7.2 Hz, 2H), 2.81 (t, *J* = 7.5 Hz, 2H) ppm; MS (ESI, *m/z*) 494 [M − H]^+^; HRMS (ESI) *m/z* calcd C_26_H_19_F_3_N_3_O_4_ [M − H]^+^ 494.1328, found 494.1332.

*5-[2-(4-Fluorophenyl)-ethyl]-3-(2-hydroxy-3-methoxyphenyl)-1-(4-trifluoromethylphenyl)-3a,6a-dihydro-1H-pyrrolo[3,4-c]pyrazole-4,6-dione* (**15e**): Pale yellow solid; ^1^H-NMR (CDCl_3_) δ 7.63 (d, *J* = 9.0 Hz, 2H), 7.53–7.46 (m, 3H), 7.03-6.98 (m, 4H), 6.83 (t, *J* = 8.7 Hz, 2H), 5.11 (d, *J* = 10.8 Hz, 1H), 4.97 (d, *J* = 10.5 Hz, 2H), 3.97 (s, 3H), 3.82 (t, *J* = 7.2 Hz, 2H), 2.87 (t, *J* = 6.9 Hz, 2H) ppm; MS (ESI, *m/z*) 526 [M − H]^+^; HRMS (ESI) *m/z* calcd C_27_H_20_F_4_N_3_O_4_ [M − H]^+^ 526.1390, found 526.1401.

*5-[2-(4-Fluorophenyl)ethyl]-3-(2-hydroxy-3-methoxyphenyl)-1-(4-methanesulfonylphenyl)-3a,6a-dihydro-1H-pyrrolo[3,4-c]pyrazole-4,6-dione* (**15f**): Pale yellow solid; ^1^H-NMR (CDCl_3_) δ 7.89 (d, *J* = 8.7 Hz, 2H), 7.54–7.46 (m, 3H), 7.00–6.96 (m, 4H), 6.84–6.78 (m, 2H), 5.19–5.16 (m, 1H), 5.04–5.00 (m, 1H), 3.96 (s, 3H), 3.81 (t, *J* = 7.8 Hz, 2H), 3.11 (s, 3H), 2.86 (t, *J* = 6.9 Hz, 2H) ppm; MS (ESI, *m/z*) 536 [M − H]^+^; HRMS (ESI) *m/z* calcd C_27_H_23_FN_3_O_6_S [M − H]^+^ 536.1292, found 536.1293.

*4-[5-[2-(4-Fluorophenyl)ethyl]-3-(2-hydroxy-3-methoxyphenyl)-4,6-dioxo-4,5,6,6a-tetrahydro-3aH-pyrrolo[3,4-c]pyrazol-1-yl]-benzenesulfonamide* (**15g**): Pale yellow solid; ^1^H-NMR (CDCl_3_) δ 7.92 (d, *J* = 9.0 Hz, 2H), 7.53–7.46 (m, 3H), 7.02–6.96 (m, 4H), 6.85–6.79 (m, 2H), 5.16–4.99 (m, 2H), 4.75 (br, 2H, NH_2_), 3.97 (s, 3H), 3.83 (t, *J* = 6.9 Hz, 2H), 2.87 (t, *J* = 7.2 Hz, 2H) ppm; MS (ESI, *m/z*) 537 [M − H]^+^; HRMS (ESI) *m/z* calcd C_26_H_22_FN_4_O_6_S [M − H]^+^ 537.1244, found 537.1252.

*5-[2-(4-Fluorophenyl)ethyl]-3-(2-hydroxy-3-methoxyphenyl)-1-phenyl-3a,6a-dihydro-1H-pyrrolo[3,4-c]pyrazole-4,6-dione* (**15h**)*:* Pale yellow solid; ^1^H-NMR (CDCl_3_) δ 7.48–7.45 (m, 3H), 7.38 (t, *J* = 7.8 Hz, 2H), 7.06–6.95 (m, 5H), 6.84 (t, *J* = 8.7 Hz, 2H), 5.05 (d, *J* = 10.5 Hz, 1H), 4.91 (d, *J* = 10.5 Hz, 1H), 3.96 (s, 3H), 3.80 (t, *J* = 7.5 Hz, 2H), 2.87 (t, *J* = 7.2 Hz, 2H) ppm; MS (ESI, *m/z*) 460 [M − H]^+^; HRMS (ESI) *m/z* calcd C_26_H_22_FN_3_O_4_Na [M + Na]^+^ 482.1492, found 482.1505.

*5-[2-(4-Fluorophenyl)ethyl]-3-(2-hydroxy-3-methoxyphenyl)-1-(4-methylphenyl)-3a,6a-dihydro-1H-pyrrolo[3,4-c]pyrazole-4,6-dione* (**15i**): Pale yellow solid; ^1^H-NMR (CDCl_3_) δ 7.48–7.45 (m, 1H), 7.36 (d, *J* = 8.4 Hz, 2H), 7.19 (d, *J* = 8.4 Hz, 2H), 7.05–6.95 (m, 4H), 6.85 (t, *J* = 9.0 Hz, 2H), 5.01 (d, *J* = 11.8 Hz, 1H), 4.91 (d, *J* = 10.5 Hz, 1H), 3.96 (s, 3H), 3.80 (t, *J* = 7.2 Hz, 2H), 2.87 (t, *J* = 7.5 Hz, 2H), 2.34 (s, 3H) ppm; MS (ESI, *m/z*) 472 [M − H]^+^; HRMS (ESI) *m/z* calcd C_27_H_23_FN_3_O_4_ [M − H]^+^ 472.1673, found 472.1680.

*5-[2-(4-Fluorophenyl)-ethyl]-3-(2-hydroxy-3-methoxyphenyl)-1-(4-methoxyphenyl)-3a,6a-dihydro-1H-pyrrolo[3,4-c]pyrazole-4,6-dione* (**15j**): Pale yellow solid; ^1^H-NMR (CDCl_3_) δ 7.43–7.39 (m, 3H), 7.02–6.85 (m, 8H), 4.93–4.92 (m, 2H), 3.96 (s, 3H), 3.83–3.78 (m, 5H), 2.87 (t, *J* = 7.5 Hz, 2H) ppm; MS (EI, *m/z*) 489 [M]^+^; HRMS (EI) *m/z* calcd C_27_H_24_FN_3_O_5_ [M]^+^ 489.1700, found 489.1693.

*1-(4-Fluorophenyl)-5-[2-(2-fluorophenyl)ethyl]-3-(2-hydroxy-3-methoxyphenyl)-3a,6a-dihydro-1H-pyrrolo[3,4-c]pyrazole-4,6-dione* (**16a**): Pale yellow solid; ^1^H-NMR (CDCl_3_) δ 7.46–7.39 (m, 3H), 7.09–6.90 (m, 8H), 4.98–4.89 (m, 2H), 3.96 (s, 3H), 3.89–3.85 (m, 2H), 2.97–2.92 (m, 2H) ppm; MS (ESI, *m/z*) 476 [M − H]^+^; HRMS (ESI) *m/z* calcd C_26_H_20_F_2_N_3_O_4_ [M − H]^+^ 476.1422, found 476.1416.

*1-(4-Fluorophenyl)-5-[2-(3-fluorophenyl)ethyl]-3-(2-hydroxy-3-methoxyphenyl)-3a,6a-dihydro-1H-pyrrolo[3,4-c]pyrazole-4,6-dione* (**16b**): Pale yellow solid; ^1^H-NMR (CDCl_3_) δ 7.49–7.40 (m, 3H), 7.10–6.96 (m, 5H), 6.85–6.47 (m, 3H), 4.99–4.92 (m, 2H), 3.96 (s, 3H), 3.83 (t, *J* = 6.6 Hz, 2H), 2.91 (t, *J* = 7.5 Hz, 2H) ppm; MS (ESI, *m/z*) 476 [M − H]^+^; HRMS (ESI) *m/z* calcd C_26_H_20_F_2_N_3_O_4_ [M − H]^+^ 476.1422, found 476.1424.

*5-[2-(4-Chlorophenyl)-ethyl]-1-(4-fluorophenyl)-3-(2-hydroxy-3-methoxyphenyl)-3a,6a-dihydro-1H-pyrrolo[3,4-c]pyrazole-4,6-dione* (**16c**): Pale yellow solid; ^1^H-NMR (CDCl_3_) δ 7.42–7.38 (m, 2H), 7.28–7.27 (m, 2H), 7.13–7.03 (m, 5H), 6.98–6.94 (m, 2H), 4.99–4.91 (m, 2H), 3.94 (s, 3H), 3.80 (t, *J* = 7.2 Hz, 2H), 2.87–2.83 (m, 2H) ppm; MS (ESI, *m/z*) 492 [M − H]^+^ ; HRMS (ESI) *m/z* calcd C_26_H_20_FClN_3_O_4_ [M − H]^+^ 492.1126, found 492.1117.

*1-(4-Fluorophenyl)-3-(2-hydroxy-3-methoxyphenyl)-5-phenethyl-3a,6a-dihydro-1H-pyrrolo[3,4-c]pyrazole-4,6-dione* (**16d**): Pale yellow solid; ^1^H-NMR (CDCl_3_) δ 7.49–7.41 (m, 3H), 7.16 (d, *J* = 6.6 Hz, 2H), 7.11–7.04 (m, 5H), 6.98–6.96 (m, 2H), 4.98–4.90 (m, 2H), 3.96 (s, 3H), 3.84 (t, *J* = 7.2 Hz, 2H), 2.90 (t, *J* = 8.1 Hz, 2H) ppm; MS (ESI, *m/z*) 458 [M − H]^+^; HRMS (ESI) *m/z* calcd C_26_H_21_FN_3_O_4_ [M − H]^+^ 458.1516, found 458.1517.

*1-(4-Fluorophenyl)-3-(2-hydroxy-3-methoxyphenyl)-5-[2-(4-methoxyphenyl)ethyl]-3a,6a-dihydro-1H-pyrrolo[3,4-c]pyrazole-4,6-dione* (**16e**): Pale yellow solid; ^1^H-NMR (CDCl_3_) δ 7.49–7.41 (m, 3H), 7.10–7.07 (m, 2H), 6.98–6.95 (m, 4H), 6.68 (d, *J* = 8.7 Hz, 2H), 4.97–4.89 (m, 2H), 3.96 (s, 3H), 3.80 (t, *J* = 6.3 Hz, 2H), 3.65 (s, 3H), 2.83 (t, *J* = 7.5 Hz, 2H) ppm; MS (ESI, *m/z*) 488 [M − H]^+^; HRMS (ESI) *m/z* calcd C_27_H_23_FN_3_O_5_ [M − H]^+^ 488.1622, found 488.1623.

The ^1^H-NMR spectra of all new compounds **6**–**16** are provided in the Supplementary Materials.

### 3.2. Biological Evaluation

#### 3.2.1. Cytotoxicty Assay 

The cytotoxicty assay was performed by 3-(4,5-dimethylthiazol-2-yl)-2,5-diphenyl tetrazoliumbromide (MTT) method. Briefly, 100 μL 4 × 10^4^ C8166 cells were seeded each well with 100 μL of different concentrations of compounds in a 96-well plate. The plate was incubated 72 h at 37 °C, 5% CO_2_ incubator. MTT (20 μL, 5 mg/mL) was added to each well and the plate was incubated at 37 °C for 4 h. Supernatant (100 μL) was discarded and 20% SDS-50% DMF (100 μL) was added. The plates were read at 570/630 nm by a Bio-Tek Elx 800 reader (Bio-Tek Instruments, Inc., Winooski, VT, USA). The 50% cytotoxic concentration (CC_50_) was calculated.

#### 3.2.2. Syncytia Inhibition Assay

The syncytia inhibition assay was applied by counting syncytia which were formed by HIV-1_IIIB_ infected C8166 cells. Briefly, HIV-1_IIIB_ supernatant and C8166 cells were seeded in a 96-well plate with different concentration of compounds. The plate was incubated in a 37 °C, 5% CO_2_ incubator for 72 h. Syncytia were counting under an inverted microscope and 50% effective concentration (EC_50_) was calculated. Therapeutic index (TI) was calculated by the ratio of CC_50_/EC_50_.

## 4. Conclusions

In summary, we have designed and synthesized a series of 3a,6a-dihydro-1*H*-pyrrolo[3,4-*c*]pyrazole-4,6-dione derivatives **6**–**16** that exhibited anti-HIV-1 activity. As expected, most of the novel compounds show single-digit micromolar antiviral potency against HIV-1. Among them, **9g** and **15i** exhibited the best overall absolute performance (EC_50_ < 5 μM, TI > 100), approximately 5- to 10- fold enhancement compared to the starting compound **6**. The structural class of agents presented herein may represent an attractive platform for developing anti-HIV-1 drugs. Further SAR studies and lead optimization are warranted to select clinically viable compound for development.

## Figures and Tables

**Table 1:**
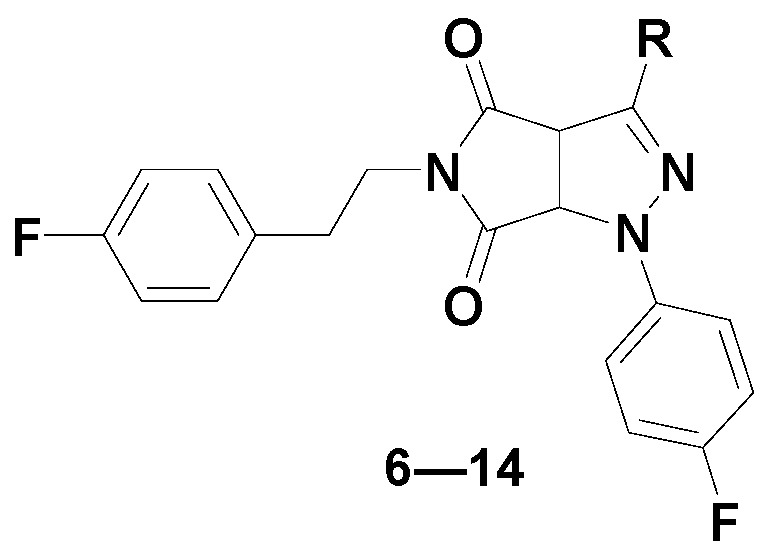
Anti-HIV-1 activity of compounds **6**–**14** in vitro ^a^.

Compd.	R	CC_50_^b^ (μM)	EC_50_^c^ (μM)	TI ^d^
**6**	2,3-OHPh	>431.92	26.39	>16.37
**7a**	2-OMe,3-OHPh	39.14	5.37	7.29
**7b**	Ph	147.64	9.21	16.03
**8**	2-OHPh	>447.35	10.11	>44.25
**9a**	2,4-OHPh	173.48	35.31	4.91
**9b**	2-OH,3-FPh	141.01	5.74	24.58
**9c**	2-OH,5-FPh	158.78	4.21	37.70
**9d**	2-OH,3-ClPh	331.51	20.42	16.23
**9e**	2-OH,3-NO_2_Ph	49.53	7.55	6.56
**9f**	2-OH,3-MePh	150.52	4.90	30.73
**9g**	2-OH,3-OMePh	>418.88	3.98	>105.26
**10a**	2-OH,4-OMePh	21.36	8.52	2.51
**10b**	2-OH,5-OMePh	188.62	4.73	39.85
**10c**	2-OH,3-OEtPh	>407.19	115.87	>3.51
**11**	2-OH,3-OMe,5-NO_2_Ph	90.29	41.94	2.15
**12a**	2,3-OMePh	>406.96	11.13	>36.56
**12b**	benzo[1,3]dioxol-4-yl	222.45	7.11	31.29
**13**	2-OH-naphthalen-1-yl	9.03	4.50	2.00
**14a**	thiazol-2-yl	158.41	35.55	4.46
**14b**	pyridin-2-yl	149.85	26.16	5.73
**AZT ^e^**		3779	0.0085	444,588

^a^ Values are means of two separate experiments; ^b^ CC_50_ (50% cytotoxic concentration), concentration of drug that causes 50% reduction in total C8166 cell number; ^c^ EC_50_ (50% effective concentration), concentration of drug that reduces syncytia formation by 50%; ^d^ In vitro therapeutic index (CC_50_ value/EC_50_ value) and ^e^ AZT was used as a positive control.

**Table 2:**
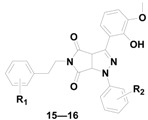
Anti-HIV activity of compounds **15**–**16** in vitro ^a^.

Compd.	R_1_	R_2_	CC_50_^b^ (μM)	EC_50_^c^ (μM)	TI ^d^
**15a**	4-F	3-F	128.89	12.08	10.67
**15b**	4-F	2-F	>418.88	112.85	>3.71
**15c**	4-F	4-Cl	166.33	25.47	6.52
**15d**	4-F	2,4-F	>403.67	65.92	>6.12
**15e**	4-F	4-CF_3_	>379.17	32.10	>11.81
**15f**	4-F	4-SO_2_Me	>372.44	65.34	>5.70
**15g**	4-F	4-SO_2_NH_2_	46.02	30.43	1.51
**15h**	4-F	H	>435.77	28.63	>15.22
**15i**	4-F	4-Me	>422.83	4.10	>103.09
**15j**	4-F	4-OMe	>408.61	21.12	>19.34
**16a**	2-F	4-F	19.58	2.52	7.78
**16b**	3-F	4-F	143.35	13.02	11.01
**16c**	4-Cl	4-F	70.13	10.43	6.73
**16d**	H	4-F	>435.73	18.87	>23.09
**16e**	4-OMe	4-F	219.37	87.15	2.52
**AZT ^e^**			3779	0.0085	444,588

^a^ Values are means of two separate experiments; ^b^ CC_50_ (50% cytotoxic concentration), concentration of drug that causes 50% reduction in total C8166 cell number; ^c^ EC_50_ (50% effective concentration), concentration of drug that reduces syncytia formation by 50%; ^d^ In vitro therapeutic index (CC_50_ value/EC_50_ value) and ^e^ AZT was used as a positive control.

## Supplementary Materials

The ^1^H-NMR spectral charts of **6**–**16** can be accessed at: http://www.mdpi.com/1420-3049/21/9/1198/s1.
