# Supplementary material for: Synthesis and Anti-HIV-1 Activity Evaluation for Novel 3a,6a-Dihydro-1H-pyrrolo[3,4-c]pyrazole-4,6-dione Derivatives
Source: Molecules. 2016 Sep 8;21(9):1198. doi: 10.3390/molecules21091198 (PMC6274355; doi:10.3390/molecules21091198)
Supplement: Supplementary file 1 [file molecules-21-01198-s001.pdf]

Supplementary Materials: Synthesis and Anti-HIV-1 Activity Evaluation for Novel 3a,6a-Dihydro-1*H*-pyrrolo[3,4-*c*]pyrazole-4,6-dione Derivatives

Guan-Nan Liu, Rong-Hua Luo, Yu Zhou, Xing-Jie Zhang, Jian Li, Liu-Meng Yang, Yong-Tang Zheng and Hong Liu

^{1}H-NMR spectra of compounds **6–16**:

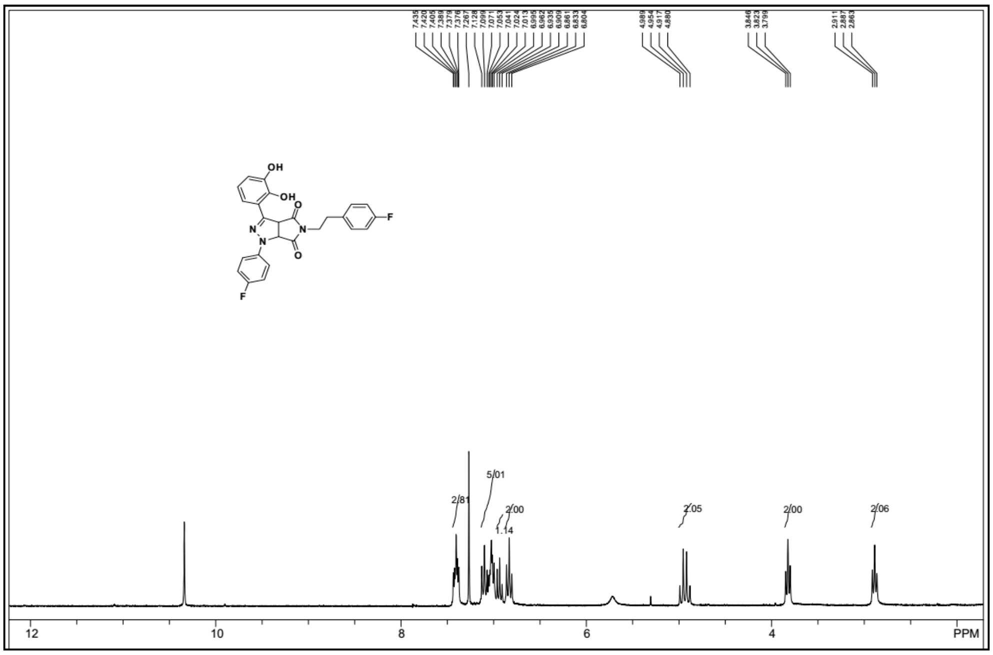

Figure S1. ^{1}H-NMR spectrum of compound **6** in $CDCl_3$.

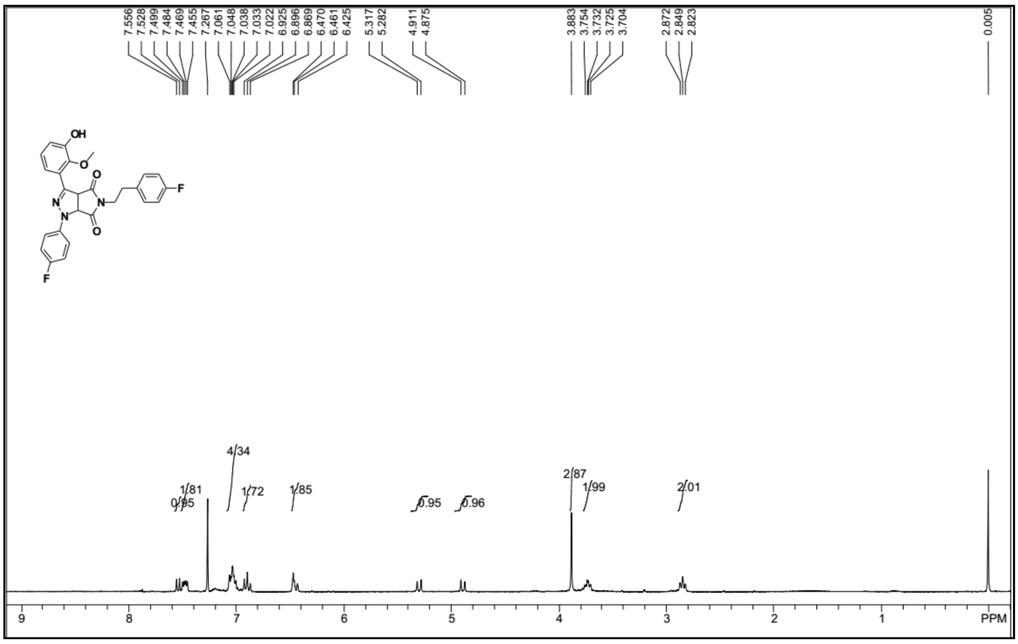

Figure S2. ^{1}H-NMR spectrum of compound **7a** in $CDCl_3$.

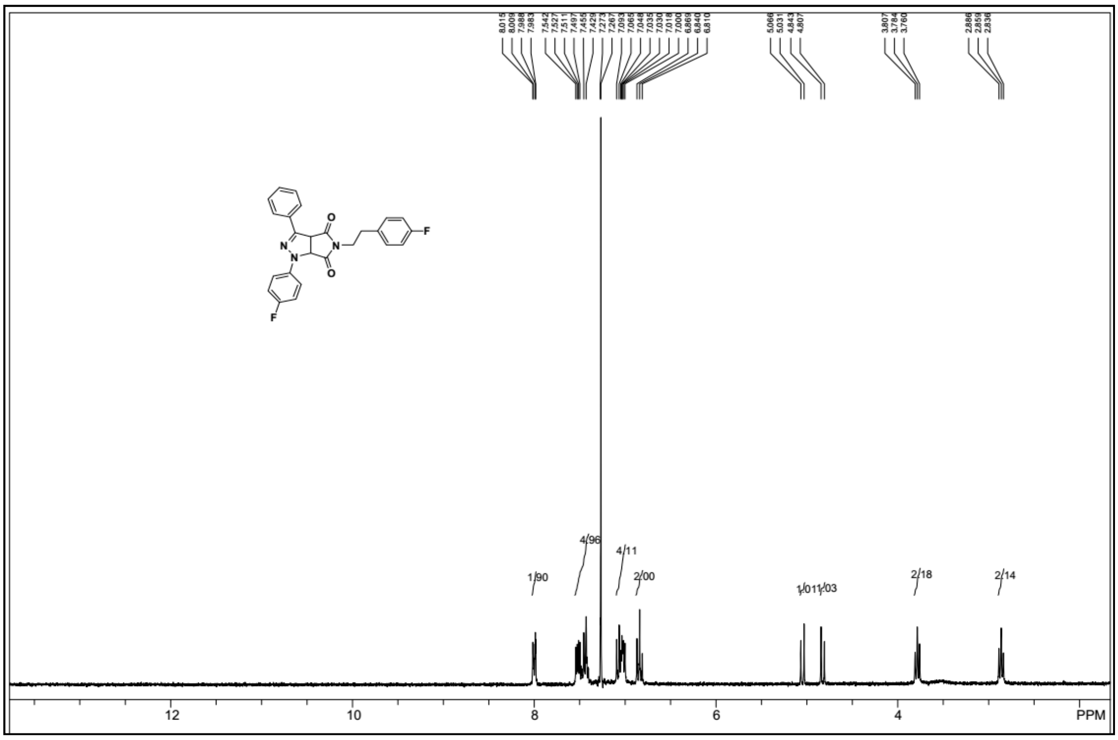

Figure S3. ^{1}H-NMR spectrum of compound **7b** in $CDCl_3$.

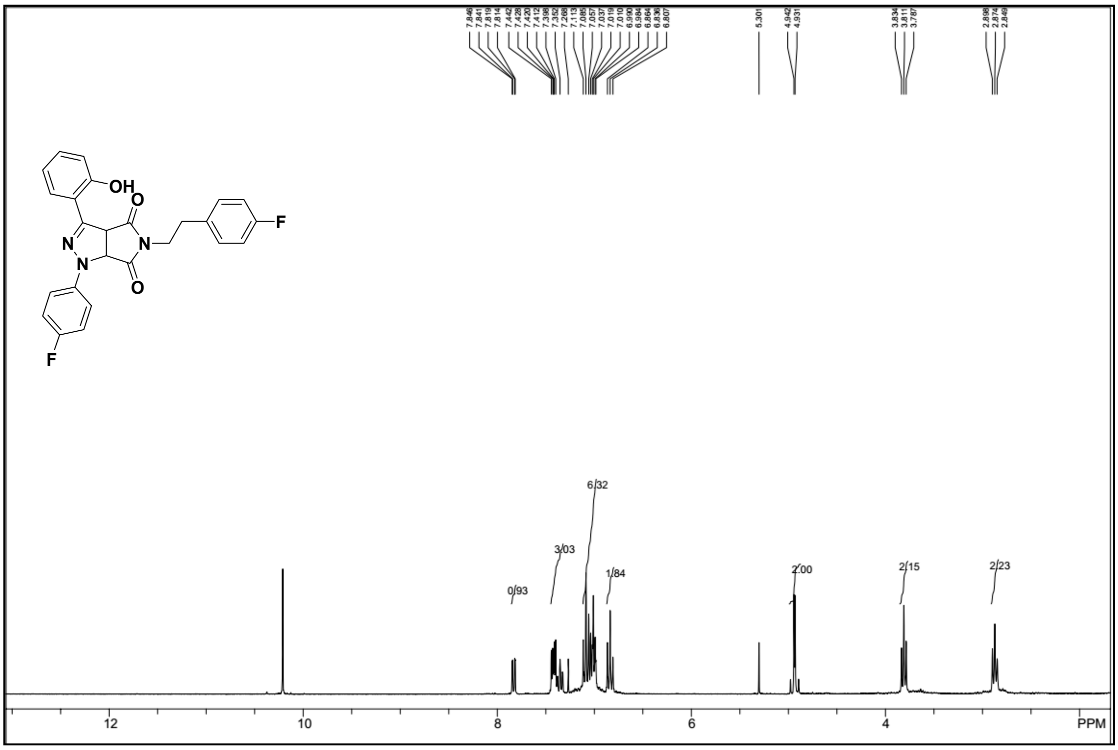

Figure S4. ^{1}H-NMR spectrum of compound **8** in $CDCl_3$.

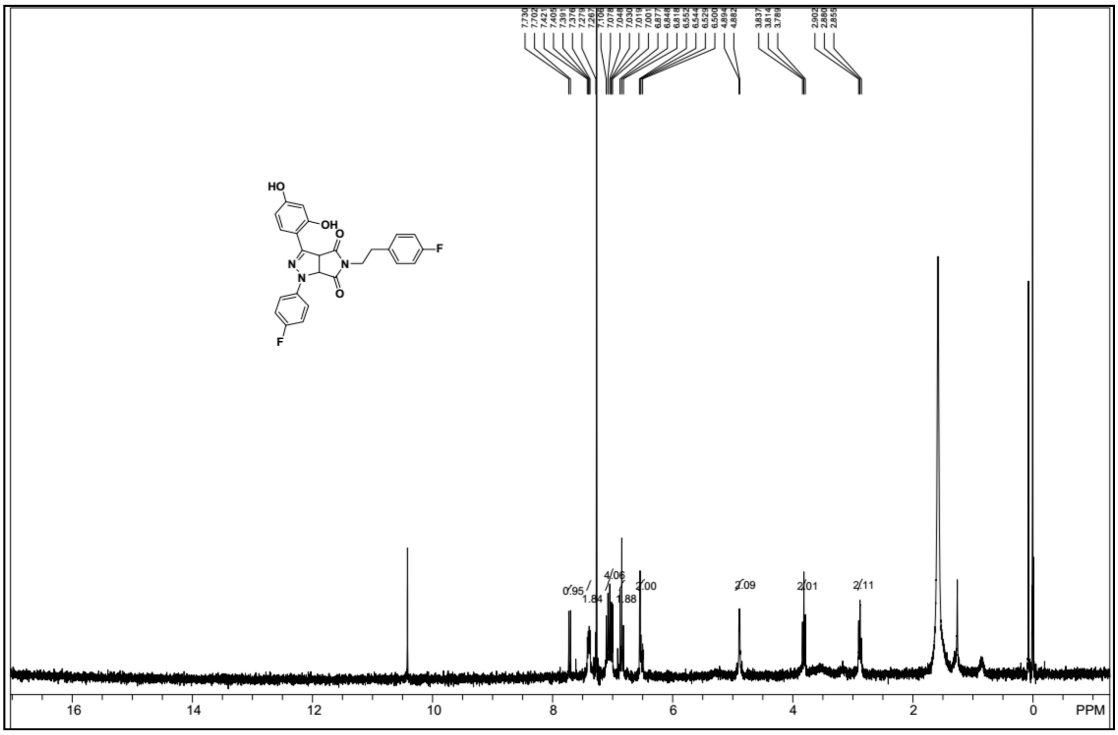

Figure S5. ¹H-NMR spectrum of compound **9a** in $CDCl_3$.

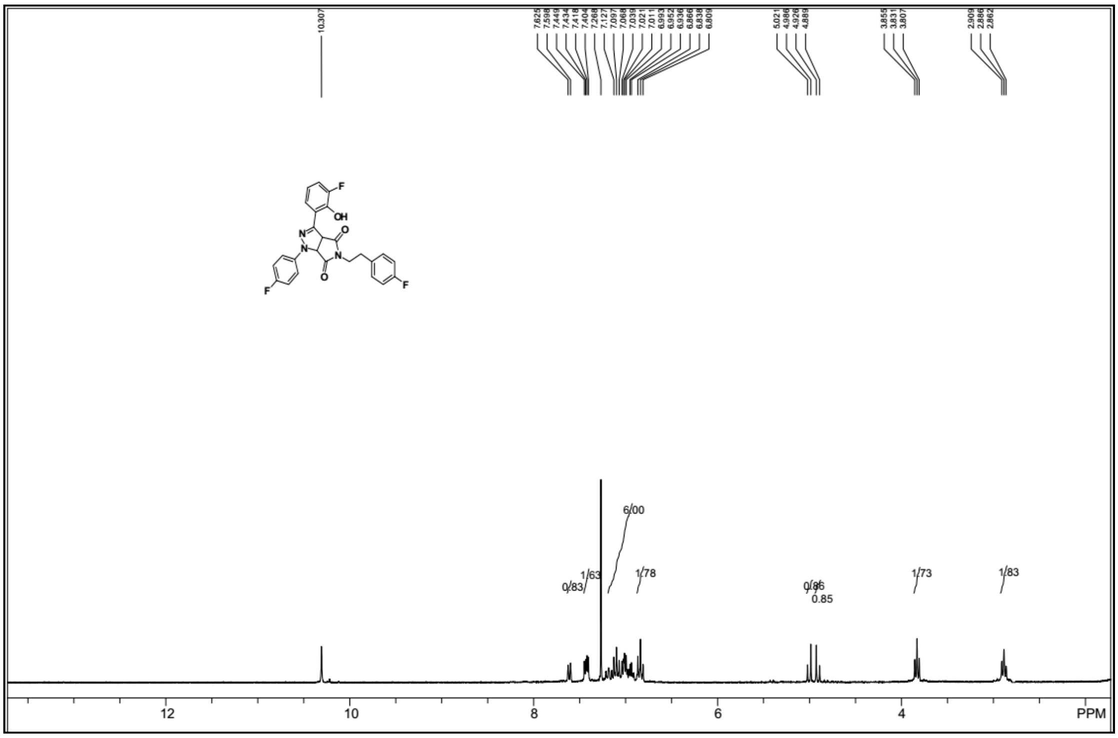

Figure S6. ¹H-NMR spectrum of compound **9b** in $CDCl_3$.

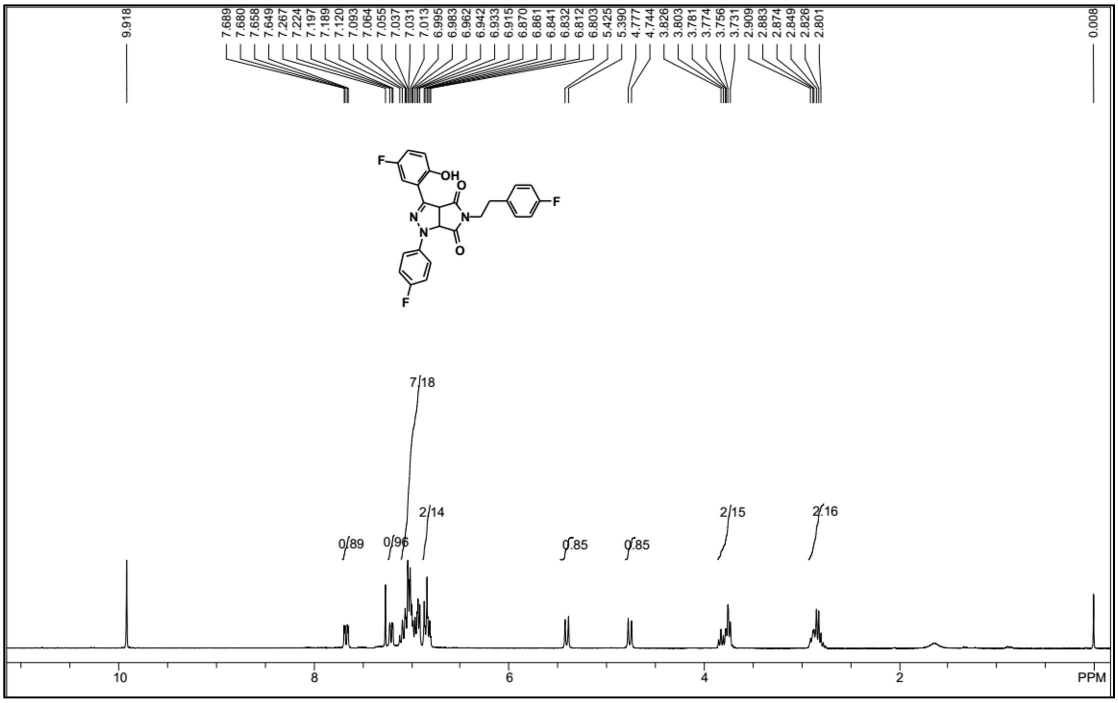

Figure S7. ^{1}H-NMR spectrum of compound **9c** in $CDCl_3$.

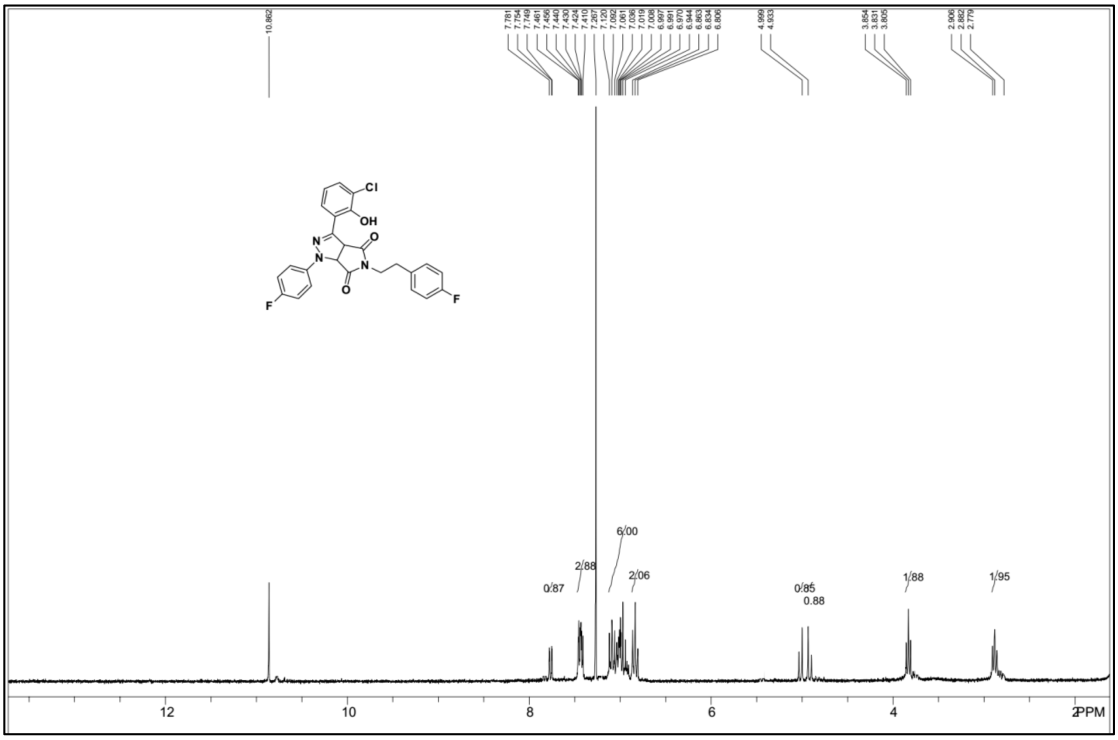

Figure S8. ^{1}H-NMR spectrum of compound **9d** in $CDCl_3$.

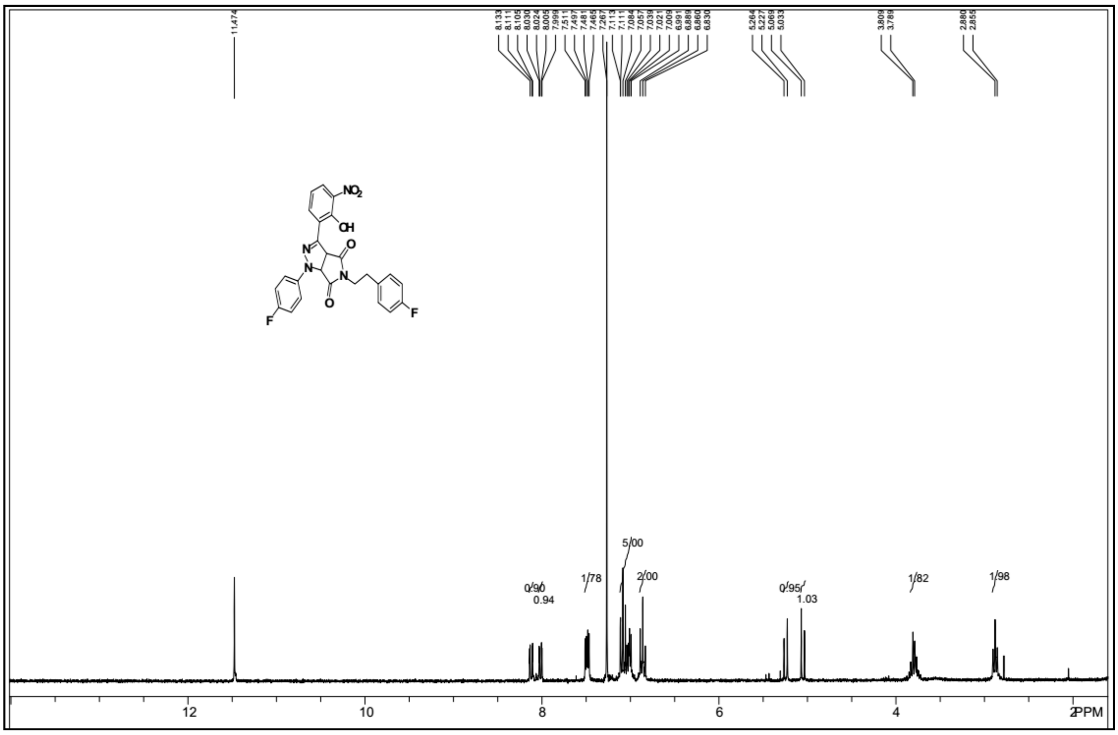

Figure S9. ¹H-NMR spectrum of compound **9e** in $CDCl_3$.

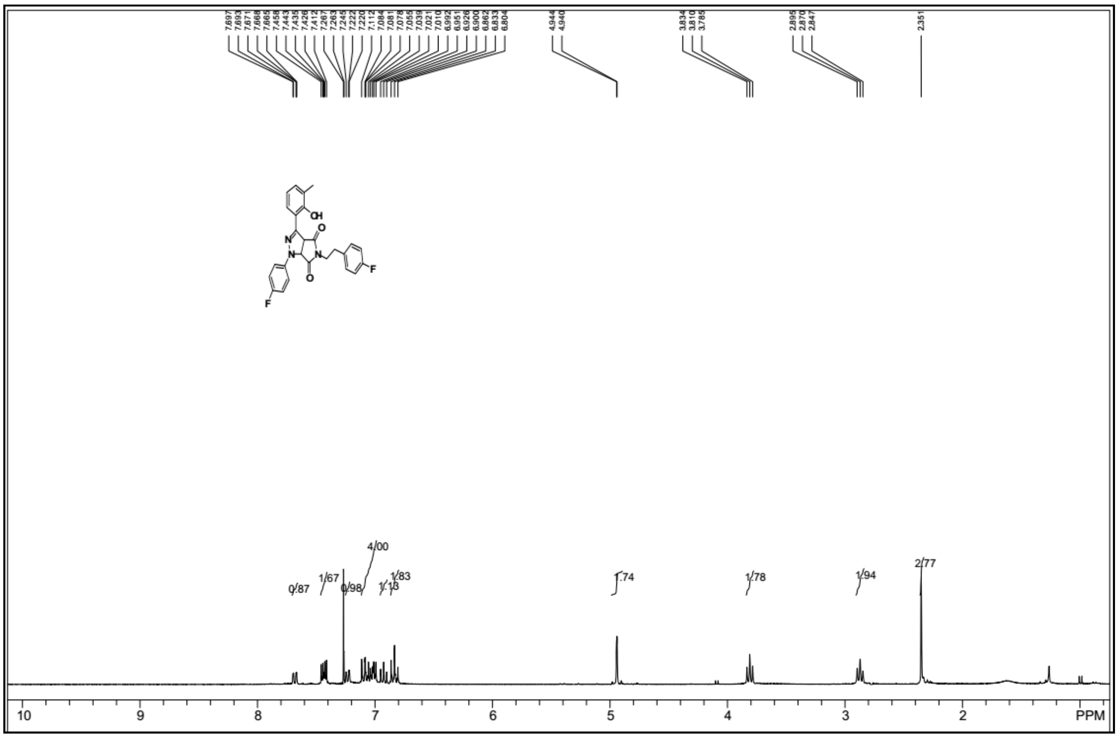

Figure S10. ¹H-NMR spectrum of compound **9f** in $CDCl_3$.

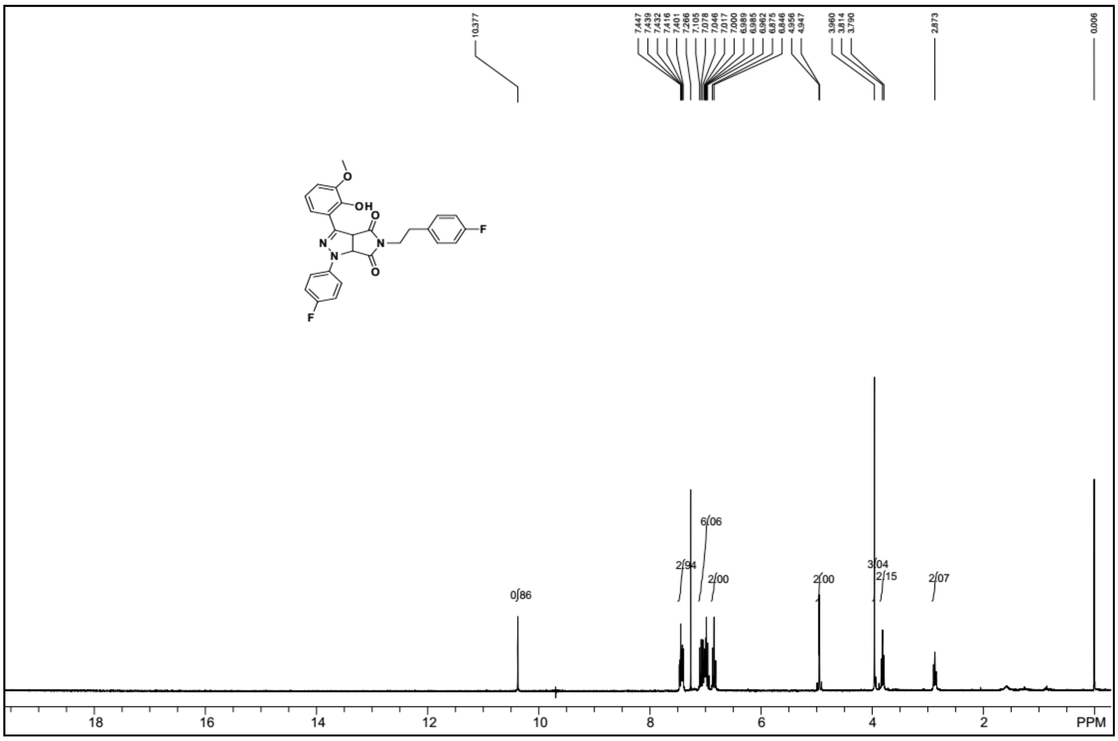

Figure S11. ^{1}H-NMR spectrum of compound **9g** in $CDCl_3$.

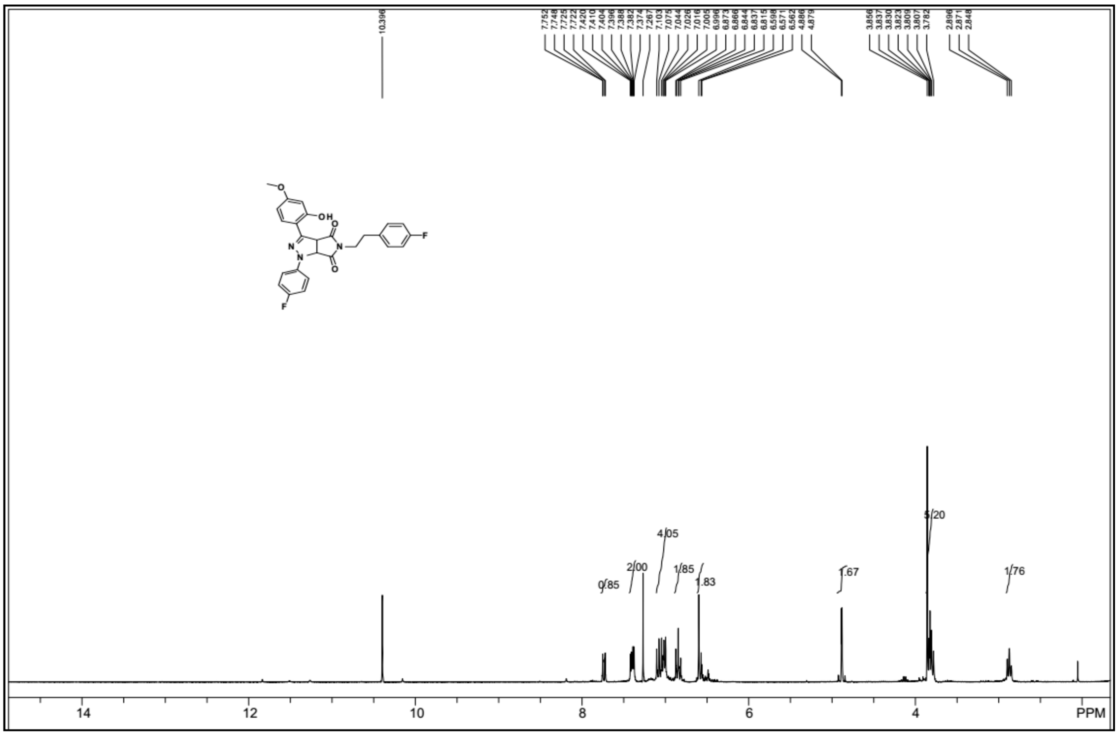

Figure S12. ^{1}H-NMR spectrum of compound **10a** in $CDCl_3$.

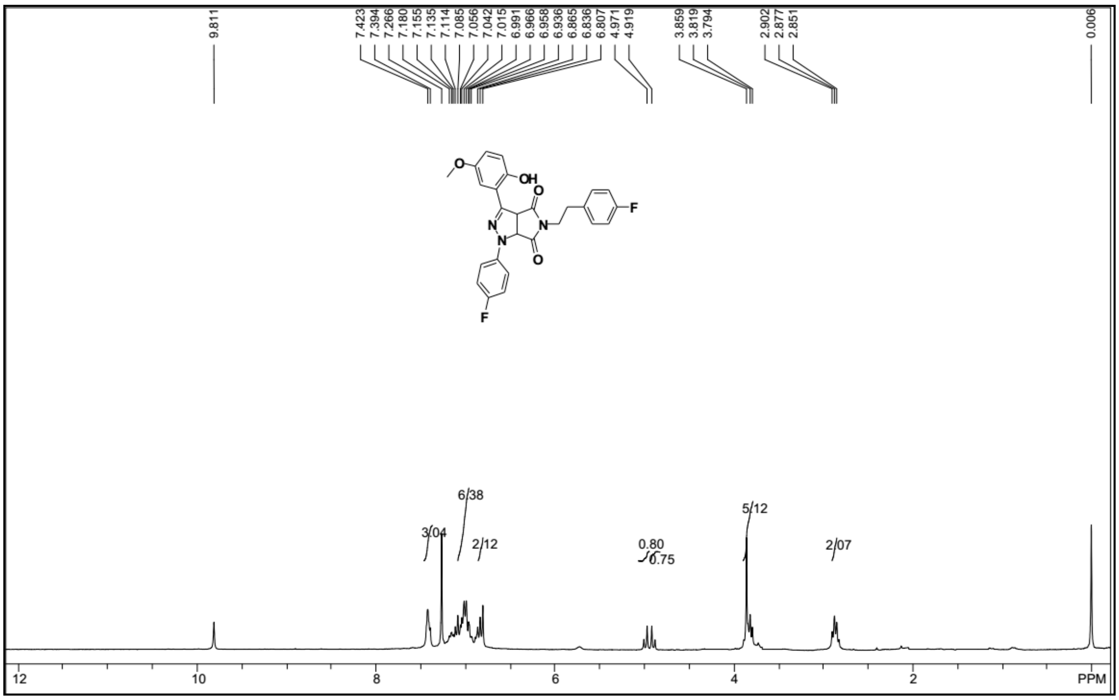

Figure S13. ^{1}H-NMR spectrum of compound **10b** in $CDCl_3$.

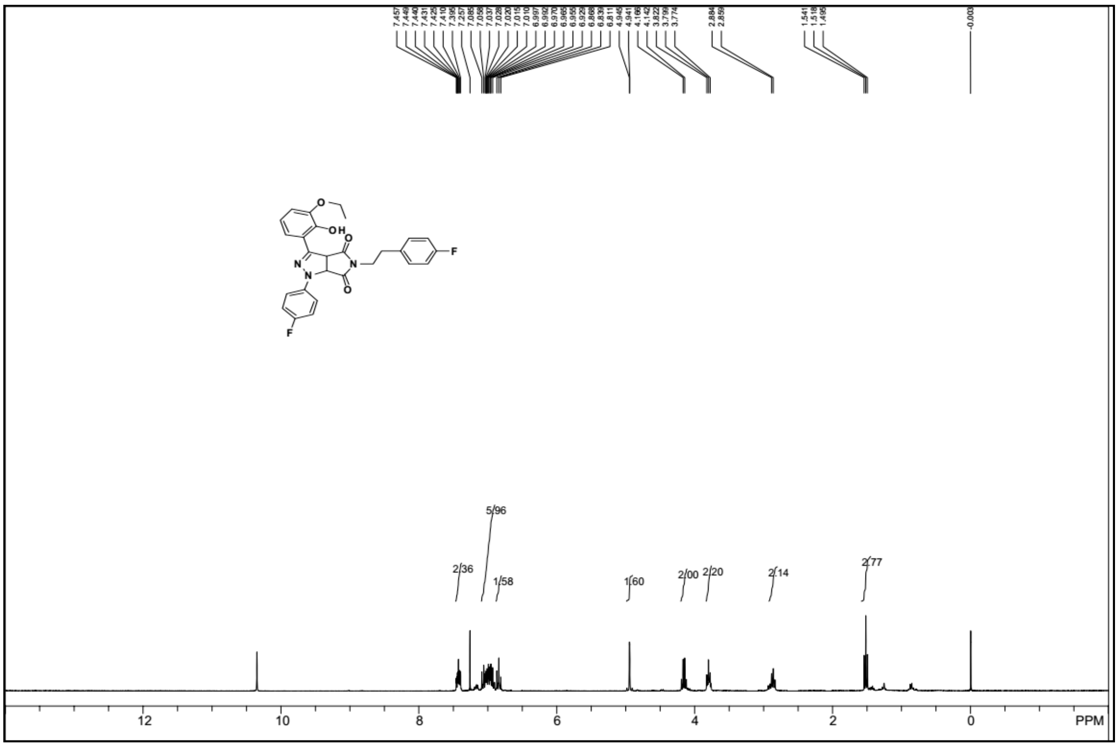

Figure S14. ^{1}H-NMR spectrum of compound **10c** in $CDCl_3$.

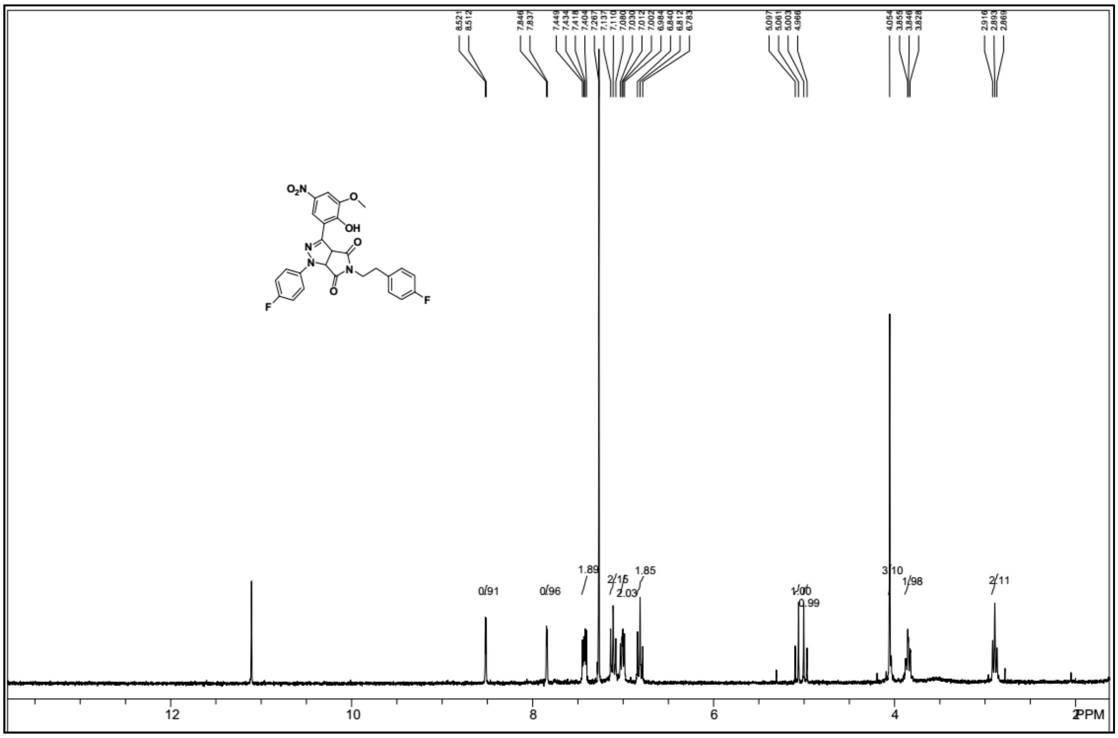

Figure S15. ^{1}H-NMR spectrum of compound **11** in $CDCl_3$.

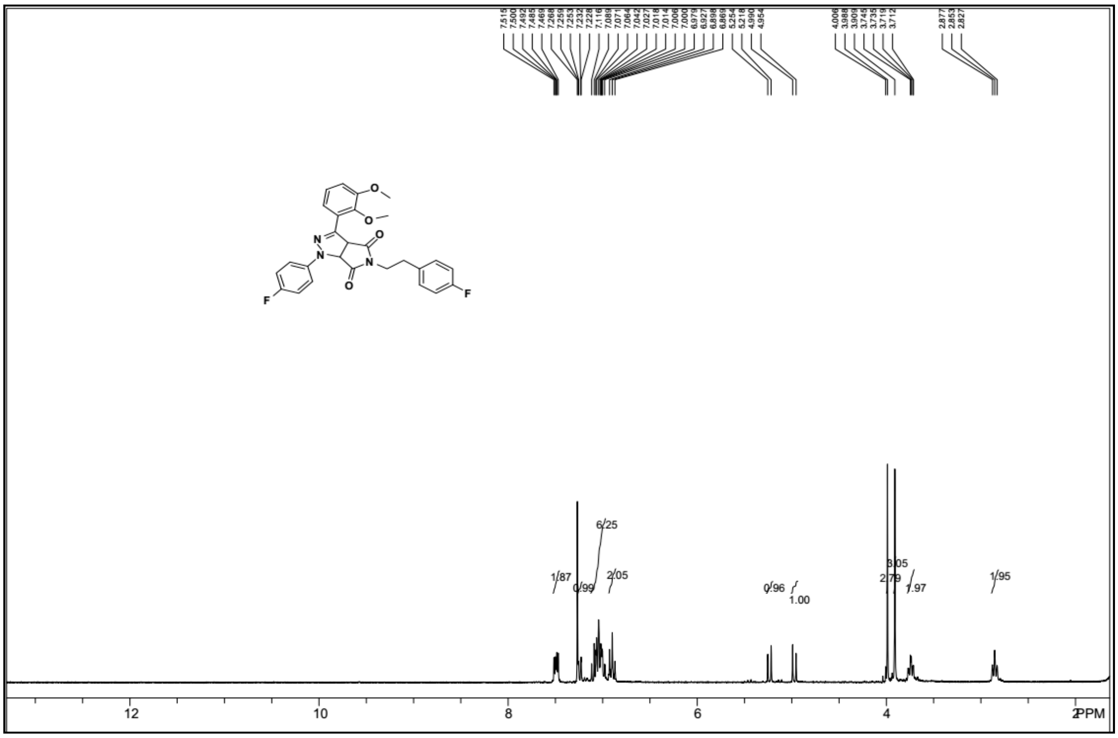

Figure S16. ^{1}H-NMR spectrum of compound **12a** in $CDCl_3$.

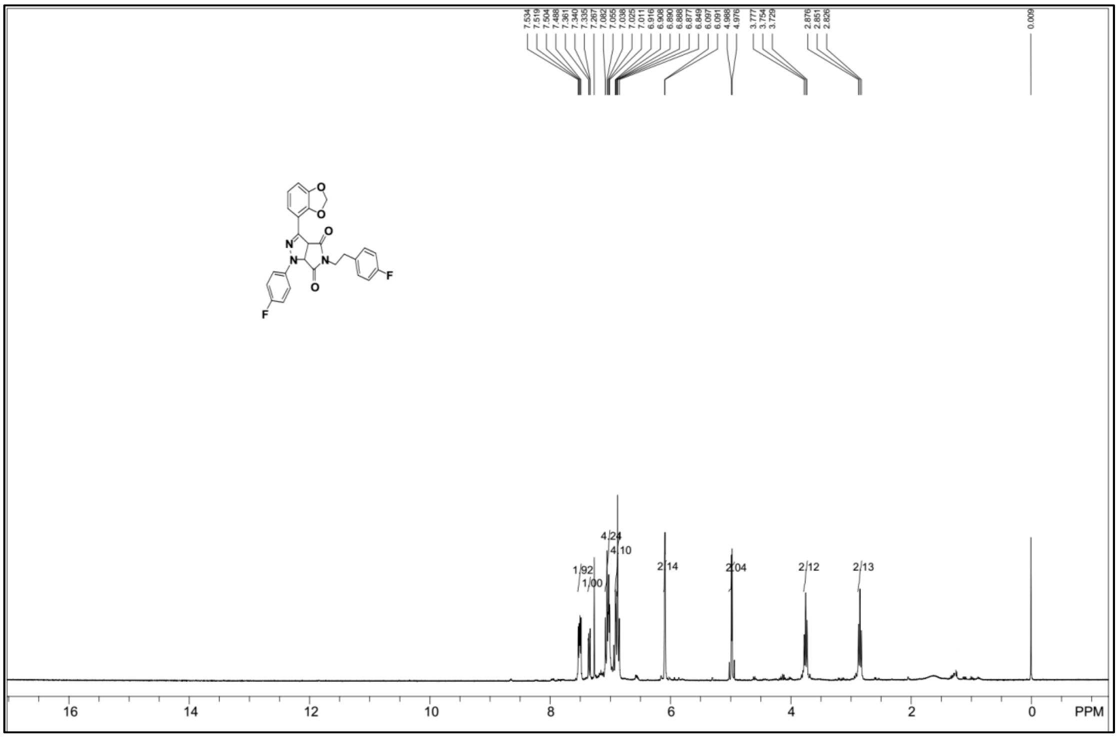

Figure S17. ^{1}H-NMR spectrum of compound **12b** in $CDCl_3$.

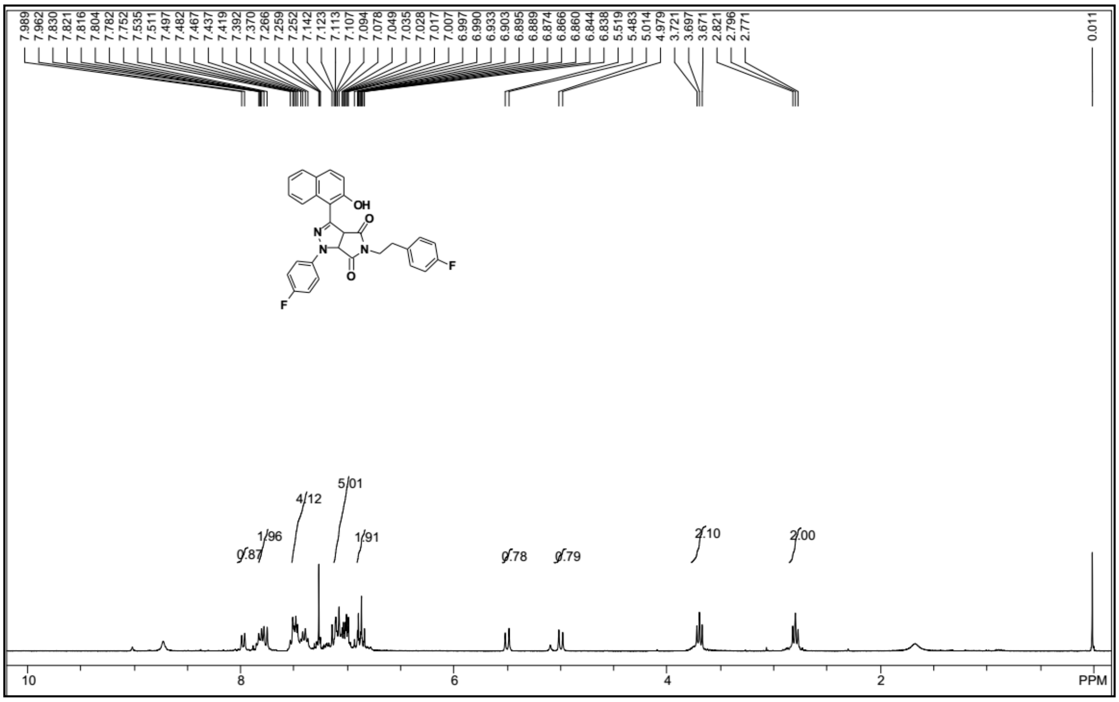

Figure S18. ^{1}H-NMR spectrum of compound **13** in $CDCl_3$.

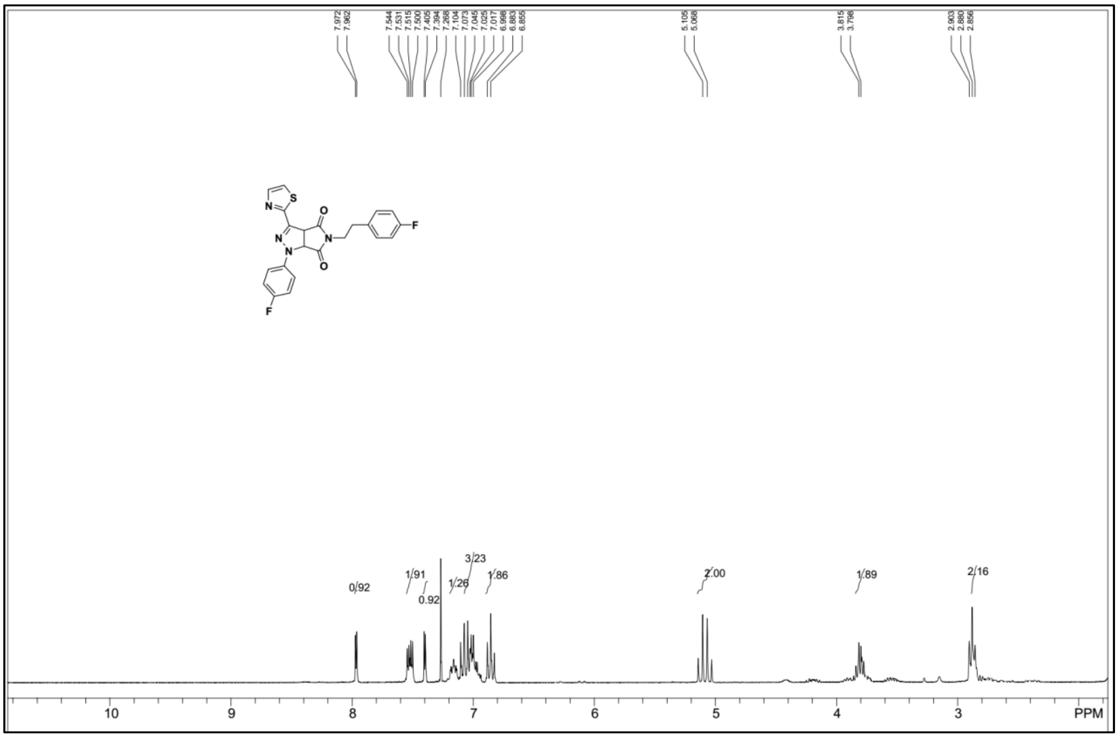

Figure S19. ^{1}H-NMR spectrum of compound **14a** in $CDCl_3$.

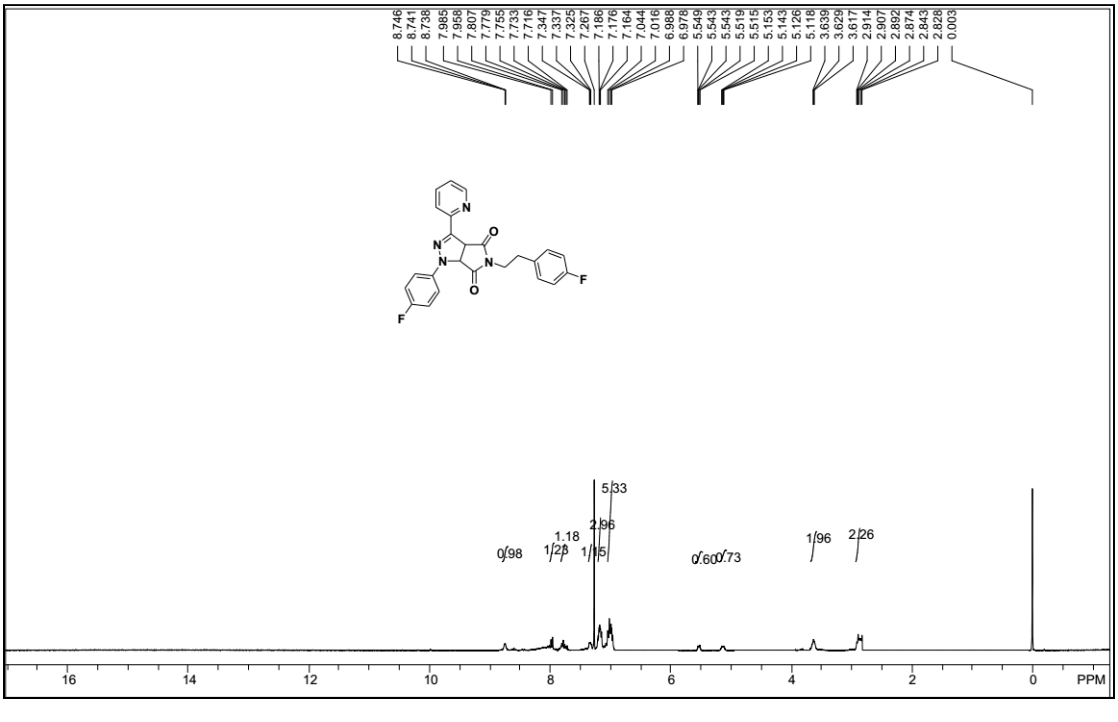

Figure S20. ^{1}H-NMR spectrum of compound **14b** in $CDCl_3$.

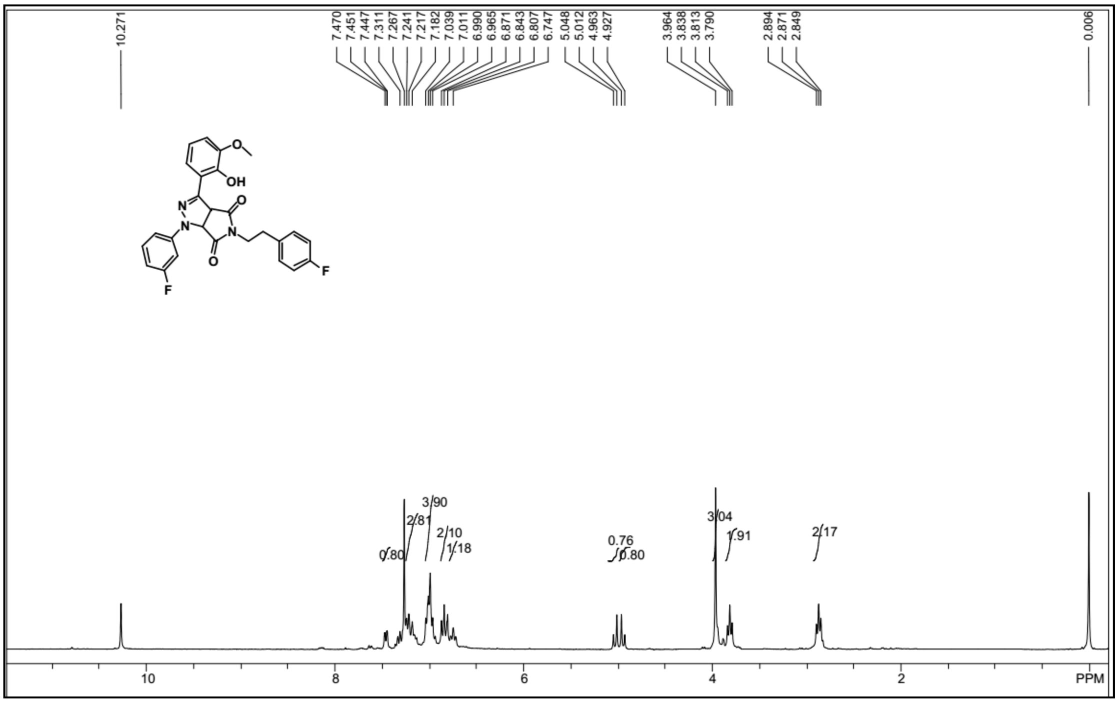

Figure S21. ^{1}H-NMR spectrum of compound **15a** in $CDCl_3$.

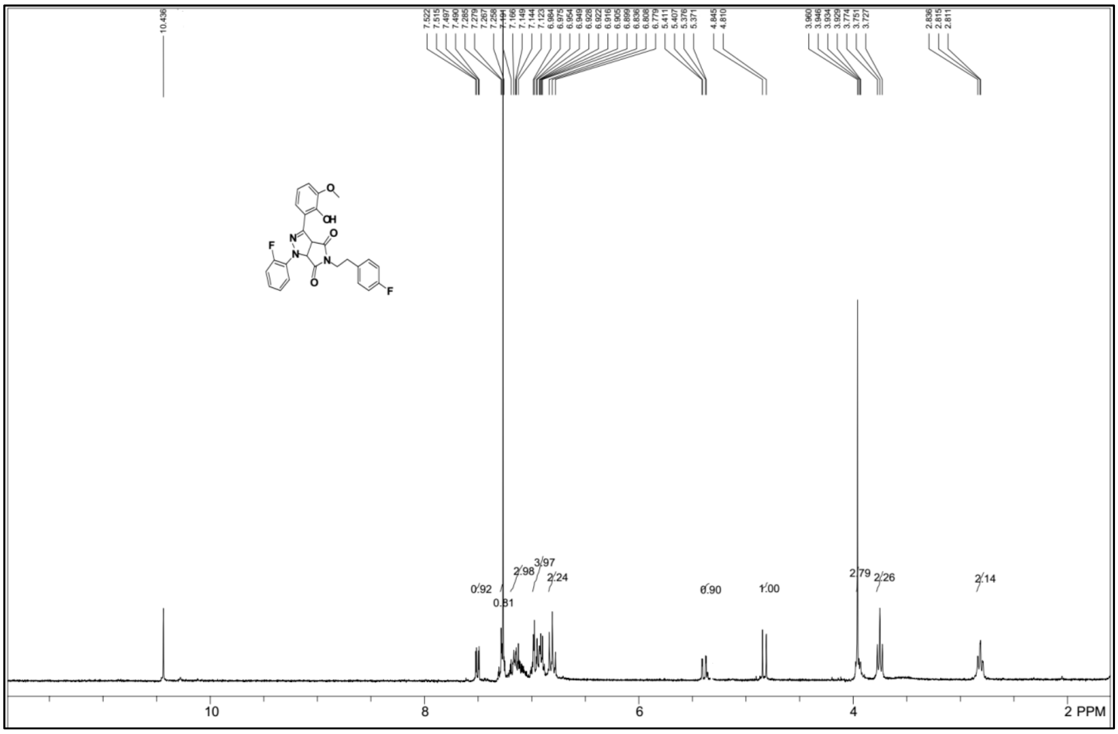

Figure S22. ^{1}H-NMR spectrum of compound **15b** in $CDCl_3$.

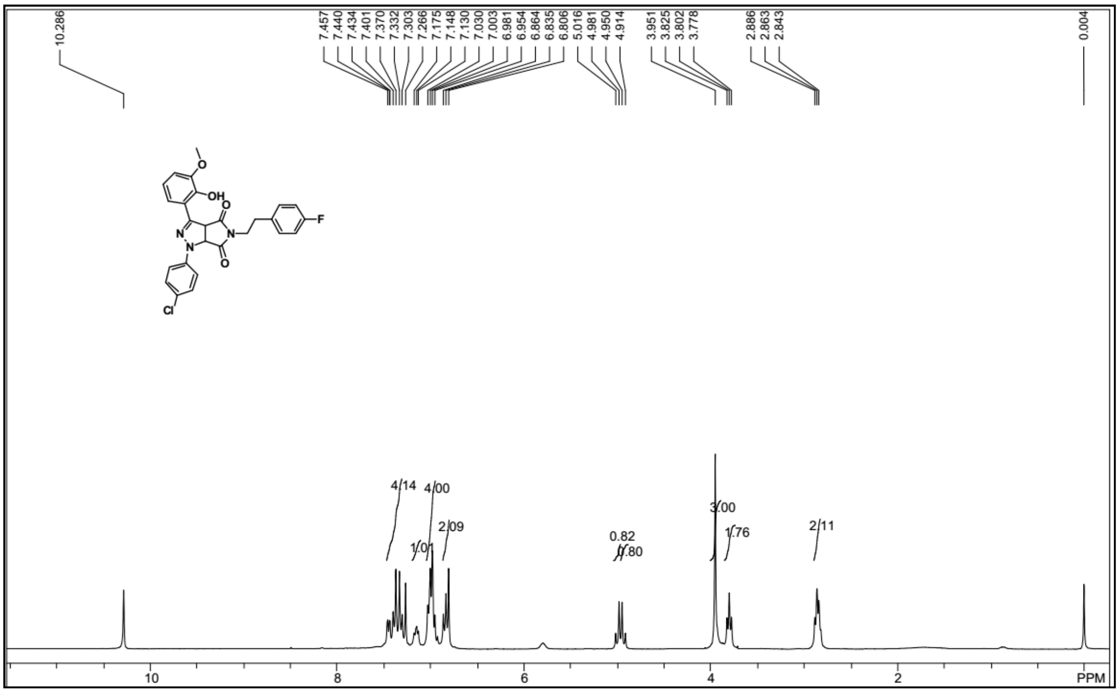

Figure S23. ^{1}H-NMR spectrum of compound **15c** in $CDCl_3$.

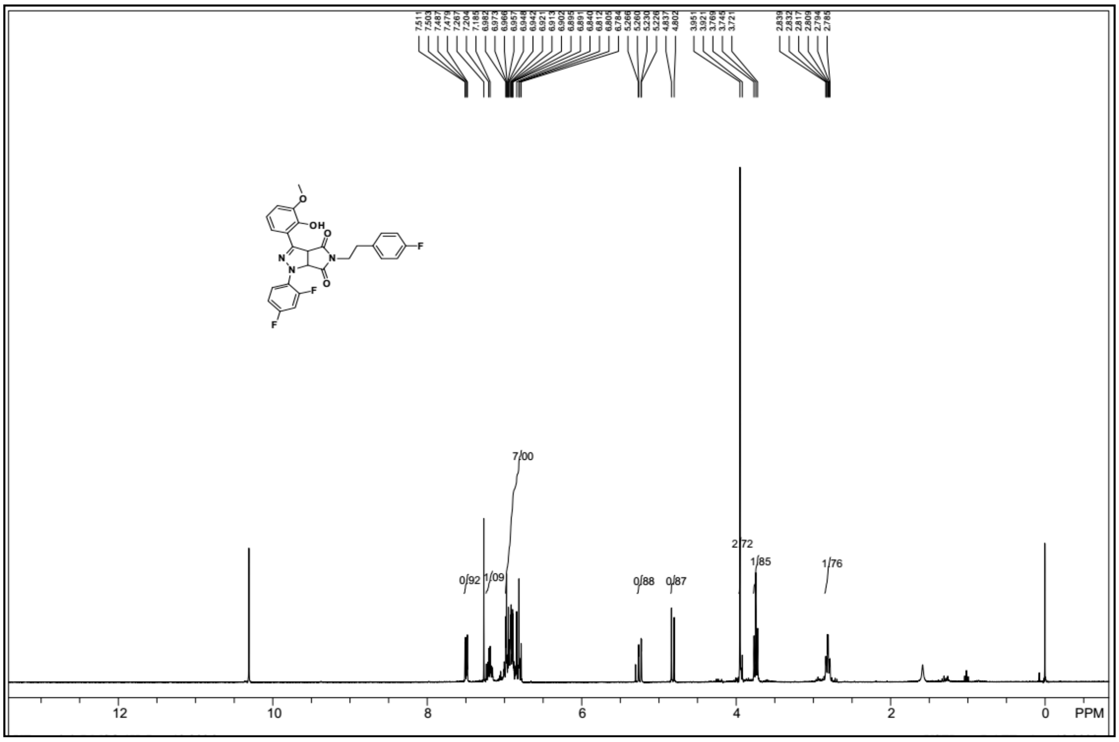

Figure S24. ^{1}H-NMR spectrum of compound **15d** in $CDCl_3$.

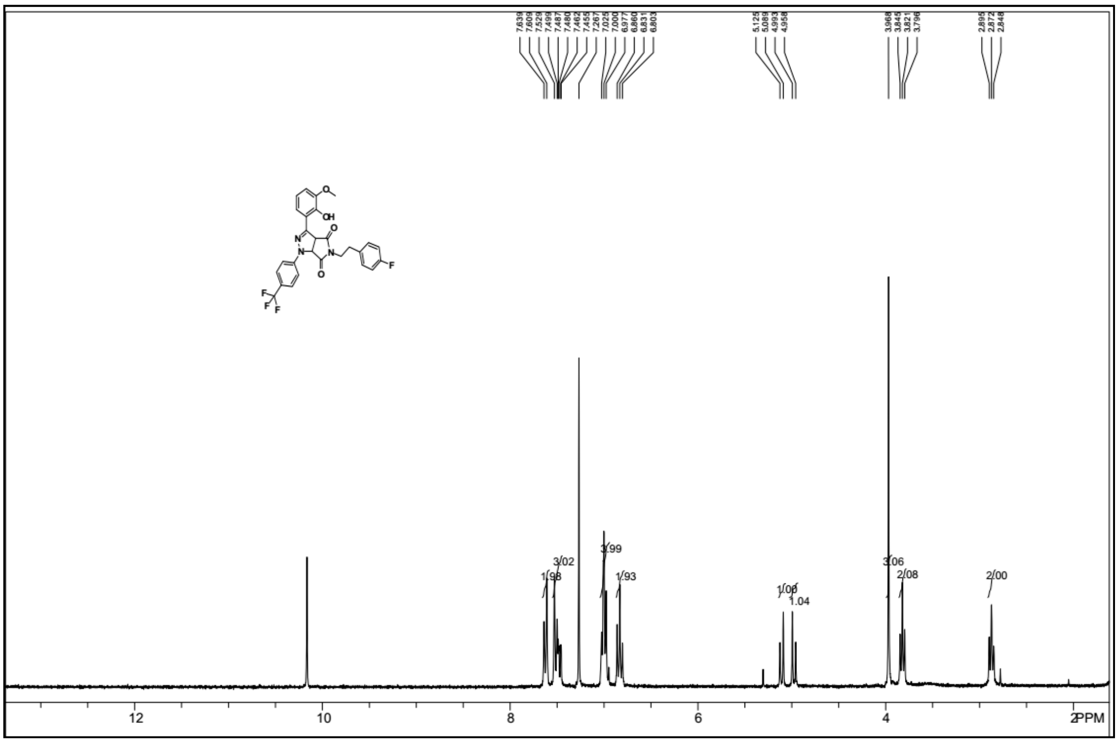

Figure S25. ^{1}H-NMR spectrum of compound **15e** in $CDCl_3$.

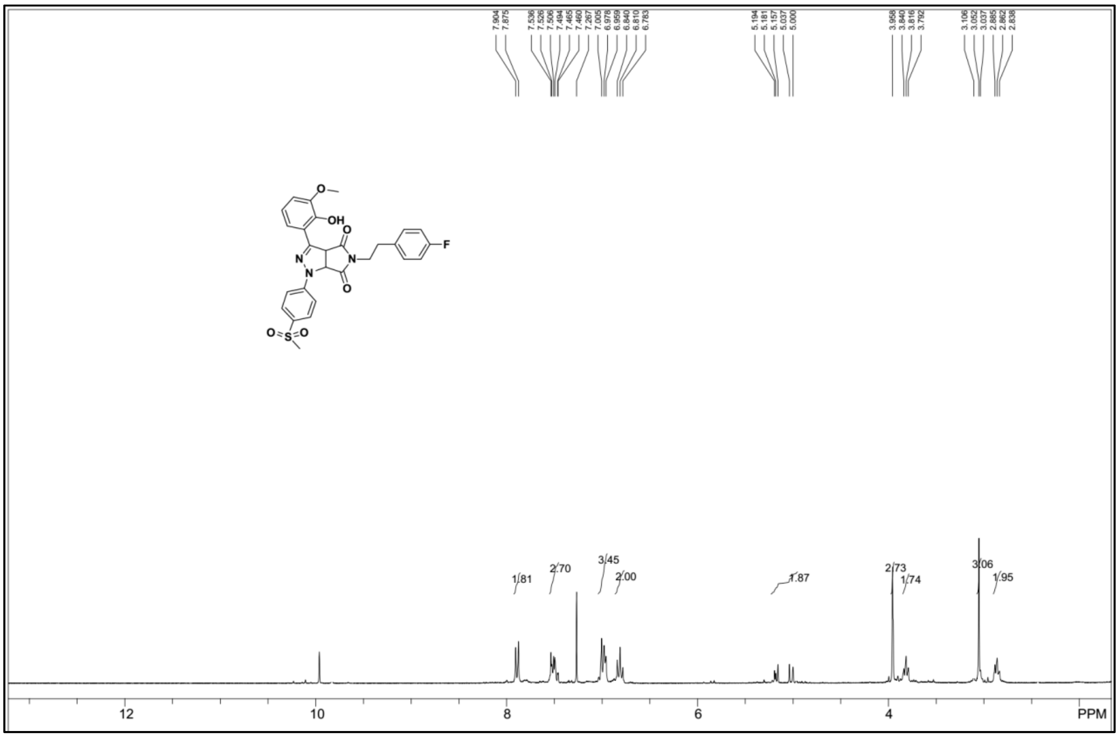

Figure S26. ^{1}H-NMR spectrum of compound **15f** in $CDCl_3$.

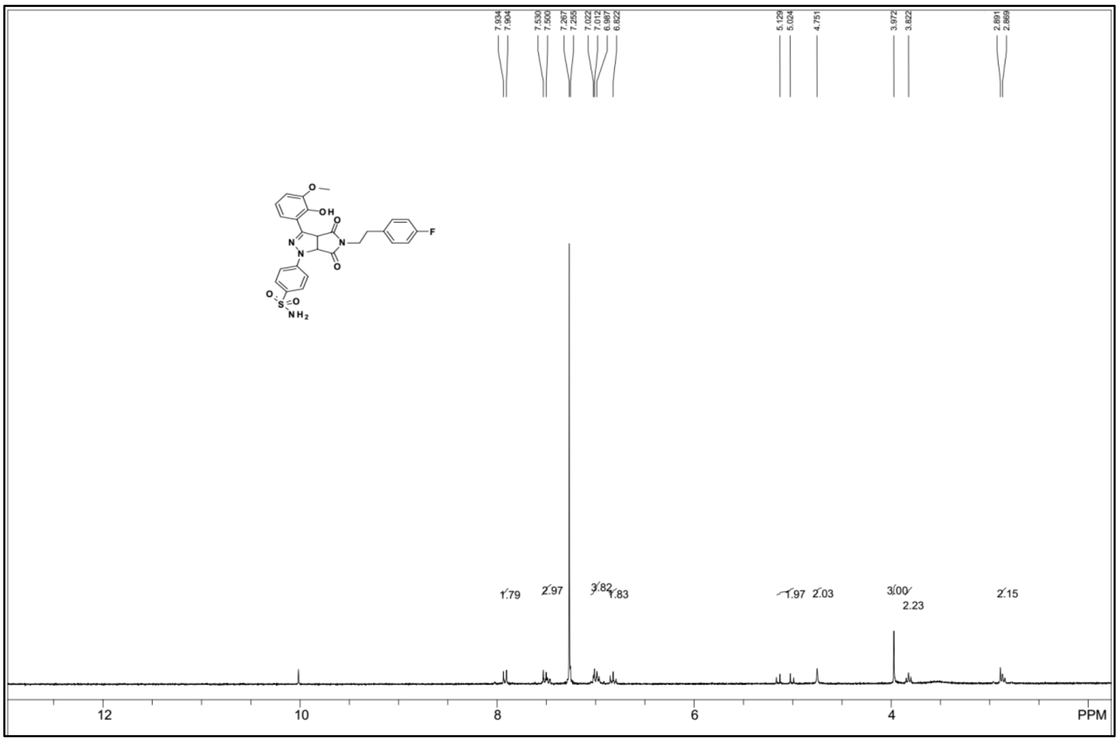

Figure S27. ^{1}H-NMR spectrum of compound **15g** in $CDCl_3$.

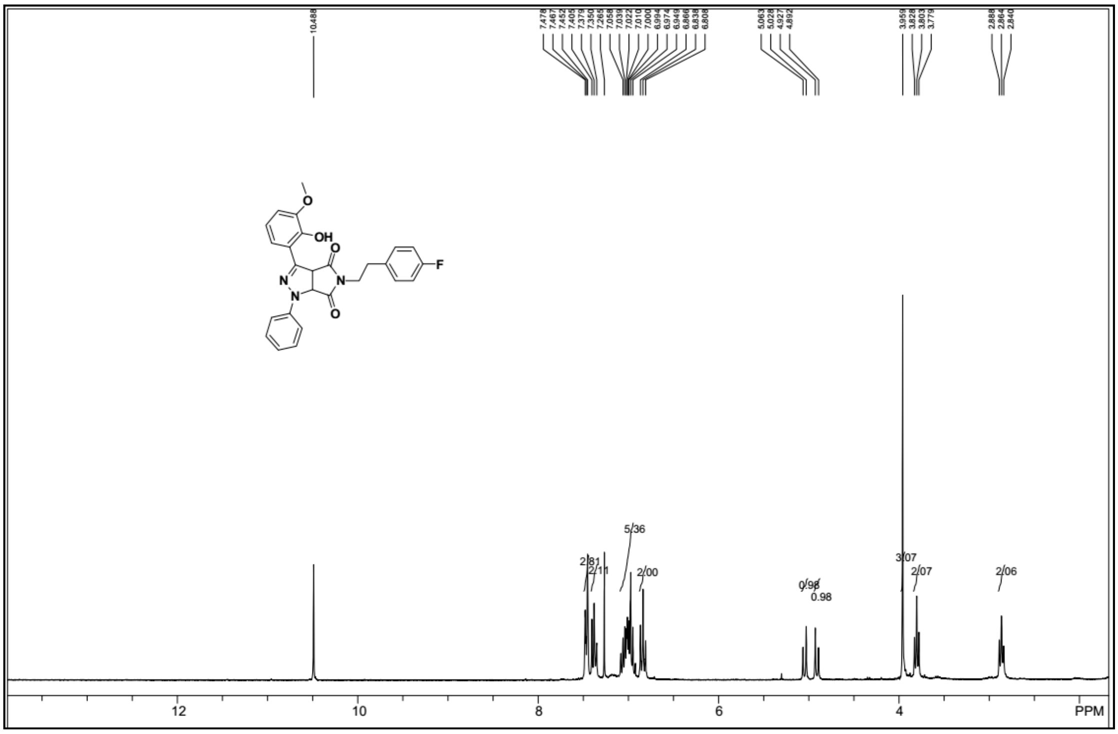

Figure S28. ^{1}H-NMR spectrum of compound **15h** in $CDCl_3$.

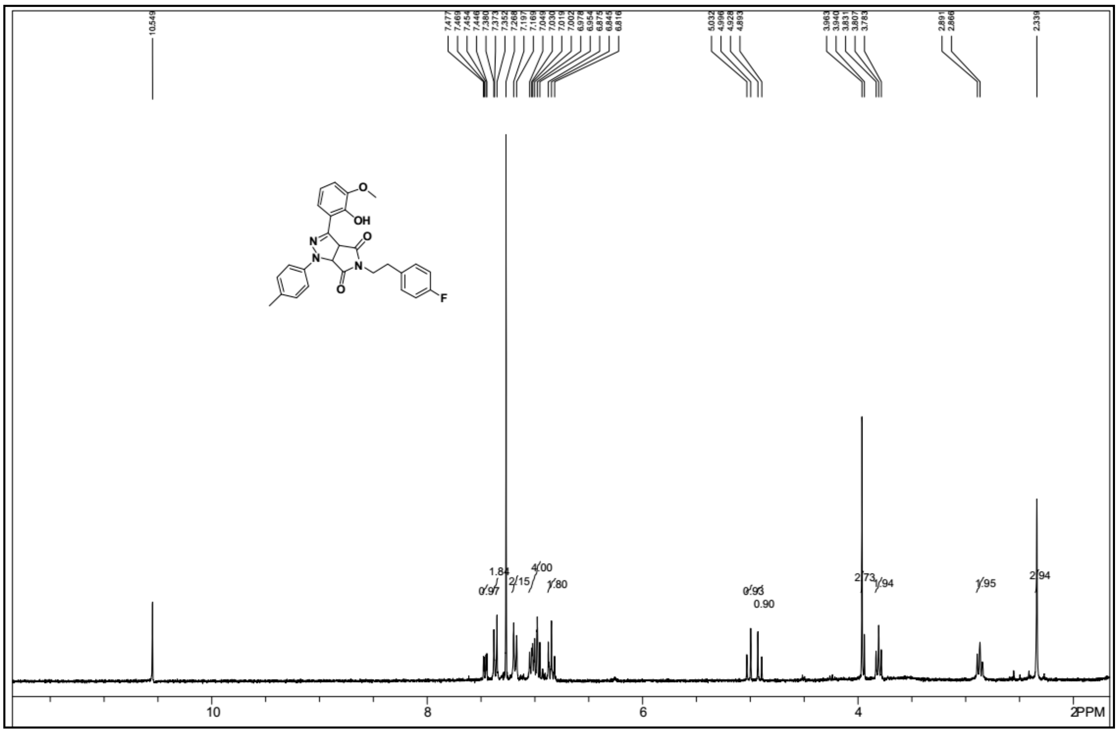

Figure S29. ^{1}H-NMR spectrum of compound **15i** in $CDCl_3$.

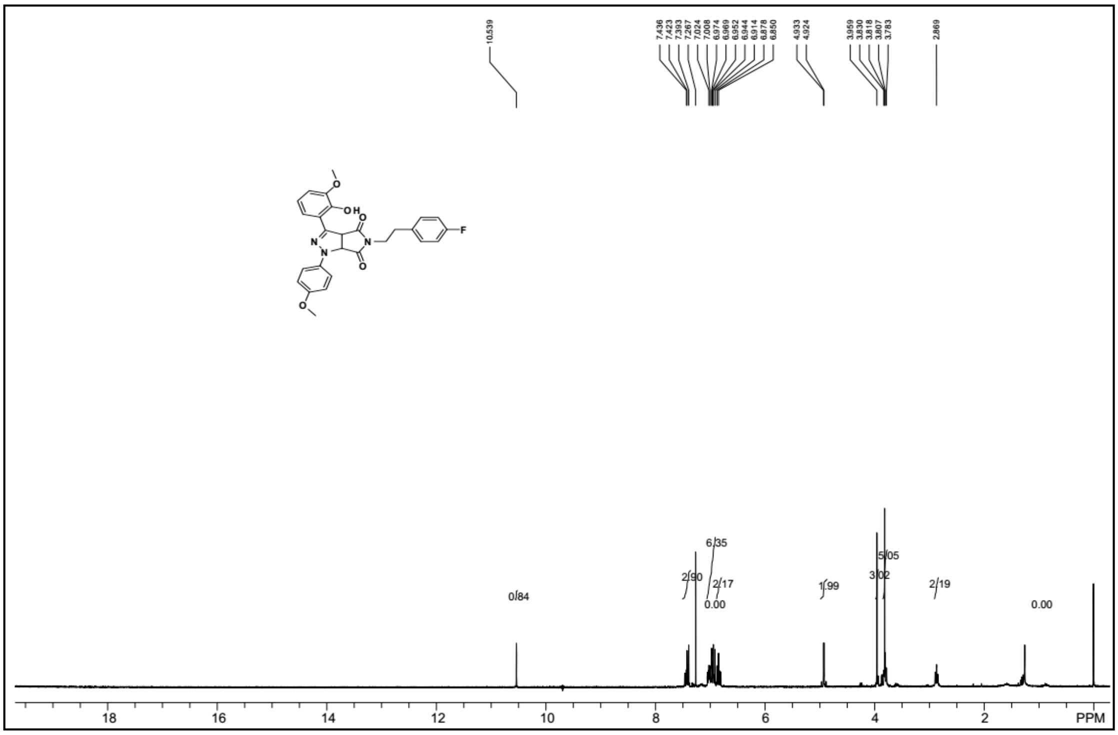

Figure S30. ^{1}H-NMR spectrum of compound **15j** in $CDCl_3$.

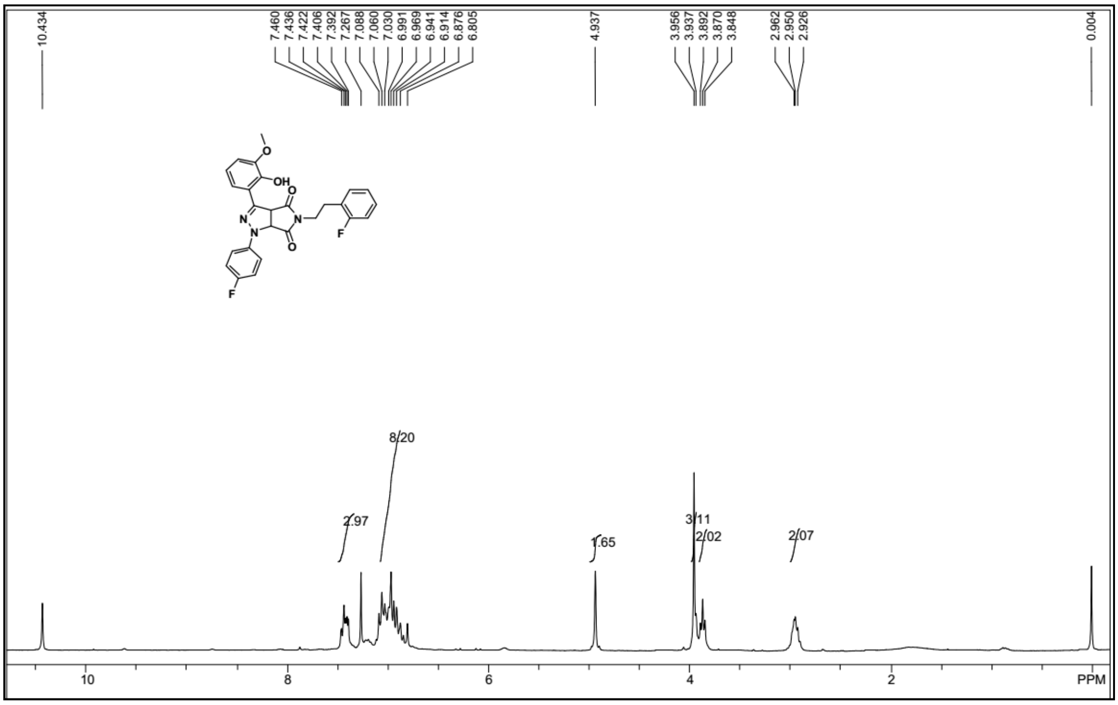

Figure S31. ^{1}H-NMR spectrum of compound **16a** in $CDCl_3$.

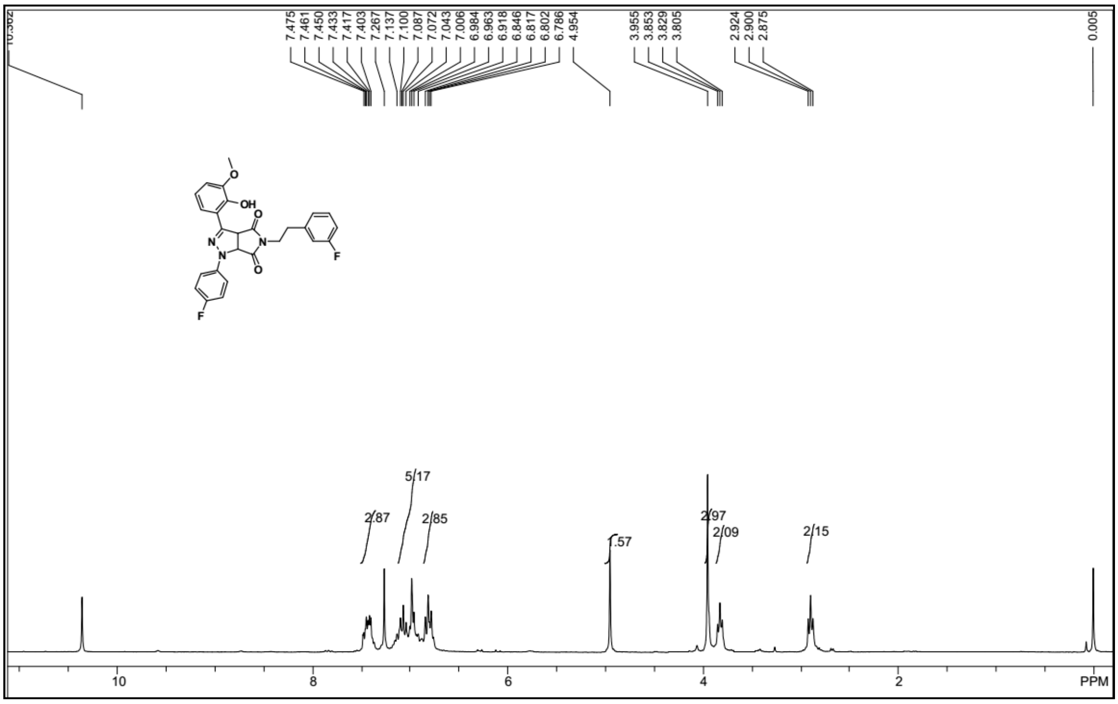

Figure S32. ^{1}H-NMR spectrum of compound **16b** in $CDCl_3$.

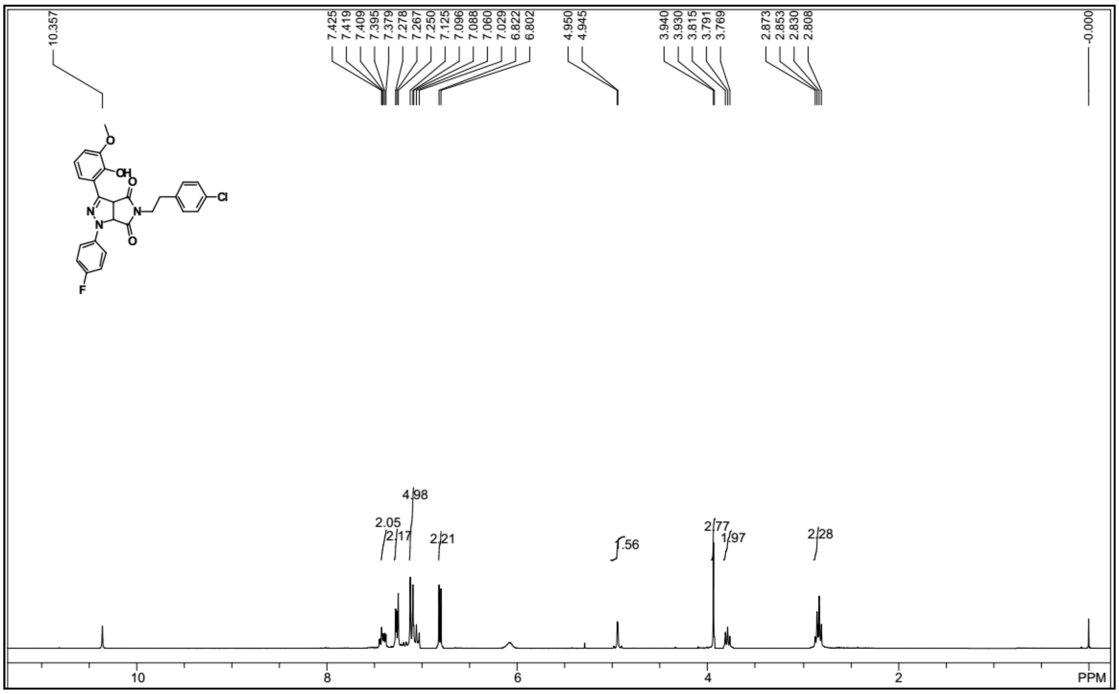

Figure S33. ^{1}H-NMR spectrum of compound **16c** in $CDCl_3$.

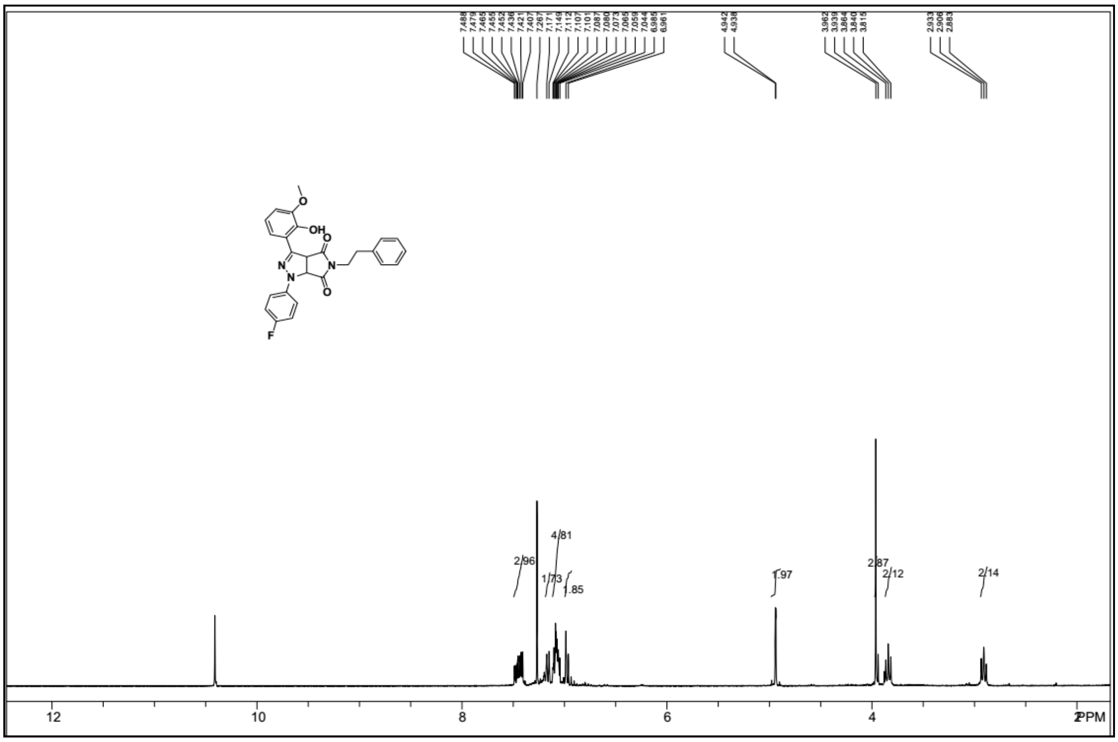

Figure S34. ^{1}H-NMR spectrum of compound **16d** in $CDCl_3$.

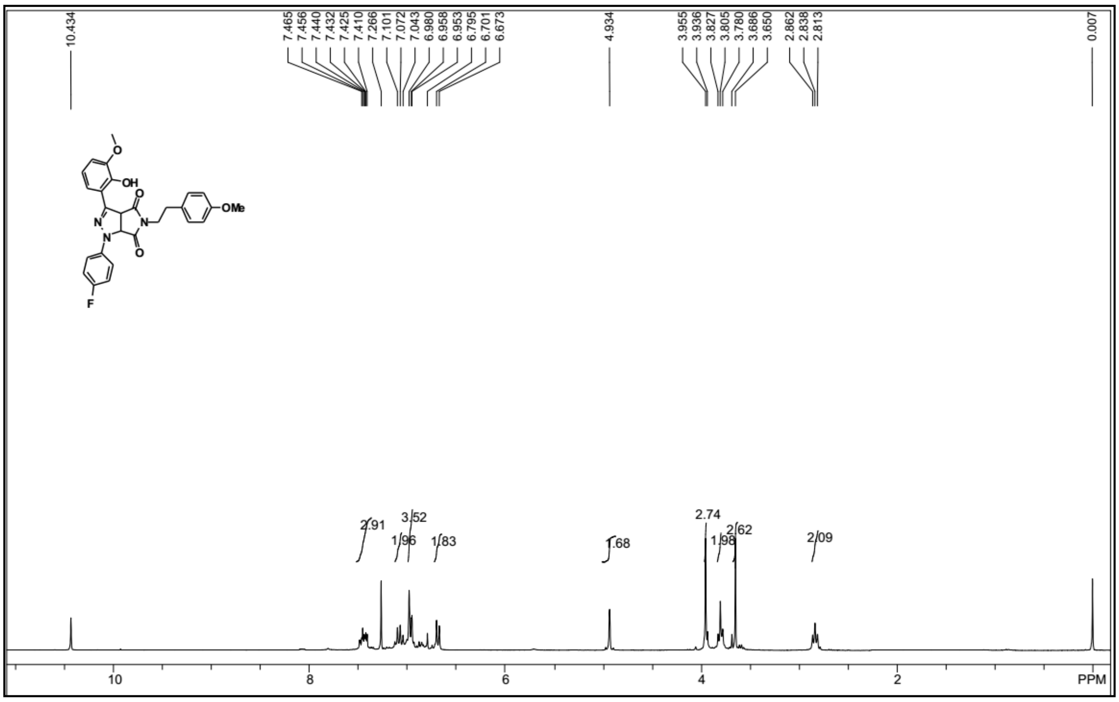

Figure S35. ^{1}H-NMR spectrum of compound **16e** in $CDCl_3$.
